# Seasonal variations in the diversity and benthic community structure of subtidal artificial oyster reefs adjacent to the Luanhe River Estuary, Bohai Sea

**DOI:** 10.1038/s41598-023-44176-6

**Published:** 2023-10-17

**Authors:** Min Xu, Yufu Xu, Jisong Yang, Jiaxing Li, Haipeng Zhang, Kaida Xu, Yunling Zhang, Takayoshi Otaki, Qi Zhao, Yi Zhang, Zengqiang Yin, Teruhisa Komatsu

**Affiliations:** 1 Key Laboratory of East China Sea Fishery Resources Exploitation, Ministry of Agriculture and Rural Affairs, Shanghai, 200090 China; 2 East China Sea Fisheries Research Institute, Chinese Academy of Fishery Sciences, No 300, Jungong Road, Yangpu District, Shanghai, 200090 China; 3 Hebei Ocean and Fisheries Science Research Institute, Qinghuangdao, 066200 China; 4 College of Fisheries and Life Science, Dalian Ocean University, Dalian, 116023 China; 5 College of Marine Science and Environment, Dalian Ocean University, Dalian, 116023 China; 6 Marine Fisheries Research Institute of Zhejiang, Zhoushan, 316021 China; 7 Hebei Provincial Technology Innovation Center for Coastal Ecology Rehabilitation, Tangshan Marine Ranching Co. Ltd, Tangshan, 063610 China; 8 Japan Fisheries Information Service Center, Tokyo, 104-0055 Japan; 9 Japan Fisheries Resource Conservation Association, Tokyo, 104-0044 Japan

**Keywords:** Biological techniques, Ecology, Zoology, Ecology, Ocean sciences

## Abstract

Artificial oyster reefs provide important spawning and nursery grounds for a variety of fishes and large mobile crustaceans. Between July 2016 and May 2017, seasonal surveys of species composition and community structure were performed in the artificial oyster reef area and control area adjacent to the Luanhe River Estuary in China. During the survey year, 56 species belonging to 50 genera, 45 families, and 19 orders were recorded. The dominant economically important fish and mobile crustaceans were *Hexagrammos otakii*, *Pholis fangi*, *Sebastes schlegelii*, *Charybdis japonica*, and *Oratosquilla oratoria*. Resident fishes belonged to the Cynoglossidae, Paralichthyidae, Pleuronectidae, and Gobiidae families. Seasonally important fish species included *Lateolabrax japonicus*, *Konosirus punctatus*, *Thryssa kammalensis*, *Hexagrammos agrammus*, and *Acanthopagrus schlegelii*. The ranges of H' values among stations were 1.18–2.16, 0.65–1.75, 1.18–2.06, and 0.62–1.92 in spring, summer, autumn, and winter, respectively. The benthic organisms present in the community of artificial oyster reef areas can be classified into groups according to month and season. The abundance biomass curves showed that the oyster reef area in spring, autumn, and winter experienced low disturbance, whereas the community structure in summer was subject to large variations from external disturbance. We also found that as the age of the oyster reefs increased, the percentage of oysters in the low shell height group (< 40 mm) decreased. The oyster density was 324 ind/m^2^ for the reef created in 2016, 724 ind/m^2^ for the reef created in 2015, and 364 ind/m^2^ for the reef created in 2013. These findings can be used to develop suitable management strategies for the sustainable maintenance of artificial oyster reef ecosystems.

## Introduction

Biological habitats, including estuarine oyster reefs, provide a variety of sustainable economic and ecological benefits to society^1–5^. However, human economic activities exert significant environmental pressures on these habitats, especially hard-substrate oyster reefs in estuarine areas^6^. Oyster reefs are one of the most important estuarine habitats, but they are the most endangered marine habitat on earth, with an estimated loss of 85% in relation to historic levels^7^. They are also the only hard substrate biological habitat in a predominately soft-sediment environment^8^. Hard substrates have been reported to attract and concentrate fishes and crustacean stocks^9^. Oysters create complex biogenic structures by growing in vertically upright aggregations, providing nursery and spawning grounds for dense assemblages of juvenile fishes and large mobile crustaceans^10^. For example, each 10-m^2^ restored oyster reef habitat can produce an additional 2.6 kg yr^−1^ of fishes and large mobile crustaceans^11^. A 1-acre oyster reef lasting 50 yrs can produce finfish valued at $40,000 dollars^12^. Additionally, oysters improve the water quality via the removal of a large quantity of particular organic matter and plankton^13^.

However, the overharvesting of wild oysters and habitat mismanagement in estuaries globally, including China, have resulted in the loss of fisheries income and the collapse of these ecologically important ecosystem engineers, and the associated ecosystem goods and services^14^. Throughout the mid-Atlantic and southeastern USA, the total biomass of the oyster *Crassostrea virginica* has been reduced to 1–2% of its historic peaks in many estuaries such as Chesapeake Bay^15^. The area (39° 10′ 36″ N, 117° 55′ 18″ E; 39° 10′ 36″ N, 117° 59′ 36″ E; 39° 07′ 30″ N, 117° 59′ 36″; 39° 07′ 30″ N, 117°5 5′ 18″ E) of living oyster reefs in the sea of Tianjin Hangu Dashentang in Bohai Bay, Bohai Sea decreased from 100 km^2^ in the 1970s to 35 km^2^ today^16^. The maximum thickness of existing reefs is only 1.2 m, with a mean value of 0.6 m^17^. The decline in oyster biomass and abundance is a consequence of the overfishing of oyster reefs, destructive fisheries practices and environmental variations^18^. These have greatly reduced the quantity and quality of reef habitat. Thus, it is necessary to perform degraded oyster reef restoration to sustainably improve and manage oyster habitats in estuarine areas^19^. In 2004, the US Army constructed a 42 ha restored oyster reef using dredged and washed oyster shells in Great Wicomico River, Chesapeake Bay^19^. Restored oyster reefs on isolated mudflats have been found to augment juvenile fish abundances and potentially increase fish productivity within estuaries^20,21^.

The Pacific oyster (*Crassostrea gigas*) is by far the most dominant oyster species, accounting for 96% of value and tonnage in the world^22–24^, and it has spread either deliberately or inadvertently to many countries^25^. Artificial structures such as artificial reefs can provide a three-dimensional habitat for colonization by fouling organisms and associated biota^26^. The accumulation of oysters and debris provides a novel habitat that can support a considerably greater biomass, richness, and density of organisms than adjacent natural habitats (e.g., *Crassostrea virginica* cages, and *Mytilus edulis* ropes)^27,28^. The Luanhe River began to runoff the mountain area at Qian’an, China in ~ 2500 years ago, and is characterized by a high sediment discharge and concentration when it enters the sea, bringing abundant nutrients and prey organisms for estuarine fishes and crustaceans^29^. The Luanhe River Estuary area of Tangshan is a historically famous fisheries ground within Bohai Bay. It is important for migration, feeding, and breeding, and serves as a nursery area for the species *Scomberomorus niphonius*, *Rhopilema esculenta*, *Acetes chinensis*, *Penaeus orientalis*, *Larimichthys polyactis*, and *Portunus. trituberculatus*, among others^30,31^. In Tangshan, China, the local fisheries community including Tangshan Marine Ranching Co. Ltd. have constructed a 2-km^2^ artificial oyster reef area through the deployment of artificial concrete reefs on the seabed, adjacent to the Luanhe River Estuary in Bohai Bay, the Bohai Sea of China^32^. This has successfully achieved sustainable annual economic outputs (ca. $230,000, unpublished commercial data) through the fishing and marine ranching (“put and take” fishery) of the sea cucumber *Apostichopus japonicas* in this area^31,32^. Recreational and sport fishing target reef fishes *Sebastes schlegelii* (6702.25 g y^−1^ and 365 ind. y^−1^) and *Hexagrannis otakii* (1430.79 g y^−1^ and 50 ind. y^−1^), and the main economic fisheries target fish *Synechogobius ommaturus* (13,122.48 g y^−1^ and 525 ind. y^−1^), were the dominant species in this artificial oyster reef area^32^. The Bohai Sea of China is an inland sea, 97,000 km^2^ in area with a 26-m mean depth, which comprises a large spawning and nursery ground for marine organisms including fishes and crustaceans^33^. In the Bohai Sea, owing to the industrial needs of sea cucumber aquaculture, many artificial reefs have been deployed^34^. These artificial reefs may develop into artificial oyster reefs because the native Pacific oyster (*C. gigas*) will gradually naturally colonize onto hard substrates^31,32^. Fan et al.^35^ verified that many buried oyster reefs distributed in the coastal plain along the northwest coast of Bohai Bay and the substrate of oyster reefs were composed of biological calcium carbonate shells . In a previous study, the ecological status of the artificial reef ecosystem was compared with that before artificial reef deployment; it was concluded that the artificial oyster system was similar to a natural rocky reef ecosystem^36^. It was also argued that seasonality is a crucial factor that needs to be carefully considered in the fisheries management of oyster reef ecosystems. Fisheries managers need to understand seasonal variations in ecosystem attributes including the community structure and food web structure of ecosystem-based management strategies before they decide on the target economic species (such as *S. schlegelii, Charybdis japonica*, and *Rapana venosa*) for specific seasons^31,32^. Furthermore, artificial oyster reefs can be regarded as an effective fisheries management tool to enhance *S. schlegelii* populations, and the present study area has also been reported to be the spawning grounds of *S. schlegelii* in early May of each year^31^. However, scientific data and related conclusions about the seasonal variations of community structure and diversity are still unreported, which restricts the understanding of the relationship between artificial oyster reef habitats and species/community characteristics. This represents an issue for the implementation of ecosystem-based artificial oyster reef sustainable management practices.

Therefore, in this study, surveys of the seasonal species composition and community structure of artificial oyster reefs and a control area adjacent to the Luanhe River Estuary were performed during the period of July 2016 to May 2017 (July 2016, September 2016, December 2016, January 2017, March 2017, May 2017, July 2017, August 2017). The aims were to: (1) identify the seasonal variations in species composition of oyster reef areas compared with the control area; (2) identify the dominant species and community diversity in different seasons and analyze seasonal variations in community structure; (3) identify the relationships between shell height groups and density of oysters in reefs of different ages. Results of this study can help fisheries managers understand whether the ecosystem function and community structure of artificial oyster reef habitats are consistent across seasons and help assess whether the current fishing policy, which varies seasonally, is reasonable and sustainable. Furthermore, the results will provide basic technical information for coastal habitat management and for the sustainable development of artificial restored oyster reefs in Bohai Bay.

## Materials and methods

### Ethical approval

Marine organism collections in the study area were permitted by the State Oceanic Administration People’s Republic of China and Tangshan Sea Ranching Industry Co. Ltd. All procedures were performed following the guidelines of the American Fisheries Society for the use of fishes and crustaceans in research^37^. The study was approved by the ethics committee of the East China Sea Fisheries Research Institute, Chinese Academy of Fishery Sciences. It did not involve endangered or protected species listed in the China Red Data Book of Endangered Animals.

### Study stations and sampling

**Figure 1:**
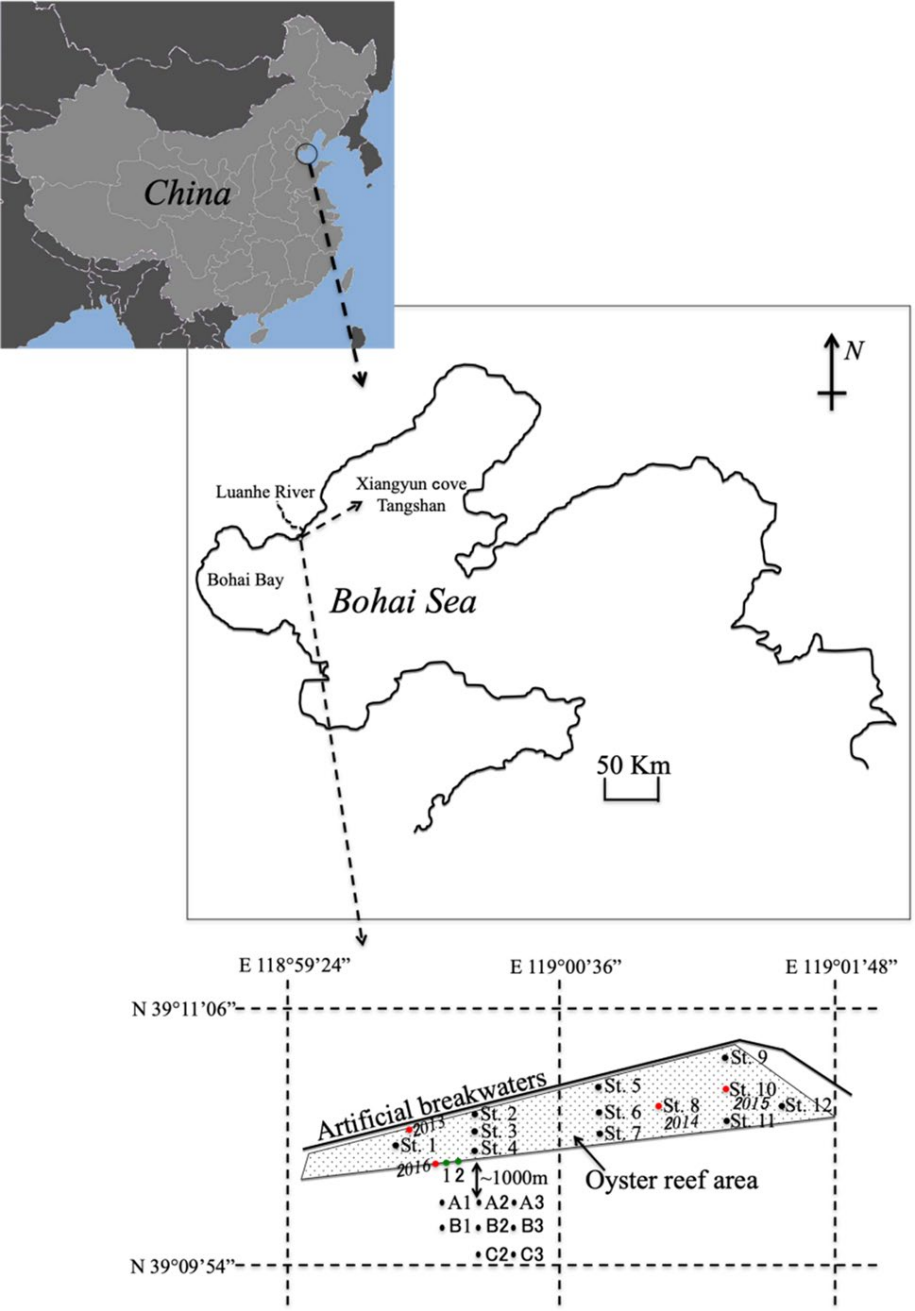
Schematic map showing the artificial oyster reef area (survey stations St.1–St.12) and control area (survey stations St.A1-St.C3) adjacent to the Luanhe River Estuary in Xiangyun Cove (Xiangyun Island, Tangshan), the northernmost part of Bohai Bay of the Bohai Sea of China. Details for the sampling stations: March 2017 (St.6-St.11), May 2017 (St.5, St.8, St.9, St.11, St.12), July 2016 (St.1, St.4, St.6-St.8), August 2017 (St.2, St.4-St.6, St.12), September 2016 (St.1-St.7, St.9), December 2016 (St.9-St.12), January 2017 (St.1-St.12), control area of July 2017 (St.A1, St.A2, St.A3, St.B1, St.B2, St.B3, St.C2, St.C3). The area (~ 2 km^2^) is denoted by a white trapezoid with black frames in the nearshore zone of the artificial reef breakwaters. The red solid dots represent the sampling stations in different reef ages created in 2013, 2014, 2015, and 2016, which were assessed by diving quadrat survey in July 2016. The green solid dots represent the sampling stations in the oyster reef experiment in May 2017.

The Luanhe River, which is 1200-km long, arises at the foot of the Yanshan Mountains and flows into Bohai Bay. The study area was adjacent to Xiangyun Cove, located near the Luanhe River Estuary, at the northeast part of Bohai Bay (Fig. 1). The study area (39° 10″14.78″–39° 10″53.86″ N, 118° 59″ 30.57″–119° 1″ 48.72″ E) is surrounded by a 4-km long, 8-m high breakwater. Since 2013, a series of stone and concrete artificial reef blocks have been deployed by the local fisheries community, which have been gradually naturally colonized by the Pacific oyster (*C. gigas*) resulting in artificial oyster reefs. The main commercial fishery target species of the local fishing community are *S. ommaturus*, *Acanthopagrus schlegelii*, *S. stigmatias*, *S. schlegelii*, Octopodidae, *C. japonica*, *R. venosa*, and *Oratosquilla oratoria*. Twelve (reef area) and nine (control area) sampling stations were established to collect marine organism samples via crab pots across a ca. 2-km^2^ artificial oyster reef area in summer (St.1, St.4, St.6-St.8 in July 12–15 2016, and St.2, St.4-St.6, St.12 in August 27–30 2017), autumn (St.1-St.7 and St. 9 in September 5–8 2016), winter (St.9-St.12 in December 1–4 2016 and St.1-St.12 in January 6–9 2017), and spring (St.6-St.11 in March 17–20 and St.5, St.8-St.9, St.11-St.12 in May 27–30 2017) and across the control area of St.A1-St.A3, St.B1-St.B3, St.C2-St.C3 in July 27–30 2017. Some of the crab pots at the stations were lost during the study, so no data were available for these stations. The length of a single crab pot was 8 m, and five were connected together at each station.

Additionally, oysters were collected by SCUBA divers, who scraped them off 0.5 × 0.5 m^2^ quadrats in July 2016. Both valves of *C. gigas* are large and concave with rippled shell layers^38^. Torigoe (1981) described them as ‘oval to spatulate’^38^, but Wakiya (1929) described one adult shell as ‘extremely elongated’^39^. We also used a ship’s crane to raise two artificial oyster reefs in May 2017 and used shovels to scrape off all sessile attached organisms. All individuals in each oyster reef were enumerated. After the surveys, fishes and crustaceans and other organisms including oysters were identified to the lowest possible taxonomic level, counted, and weighed to the nearest 0.1 g of wet weight in the laboratory.

We used a YSI multi-parameter water quality analysis measurer (EXO-2, YSI, Yellow Springs, OH, USA) to measure the water temperature (°C), salinity (ppt), pH, dissolved oxygen content (mg L^−1^), total dissolved solids content (g L^−1^), chlorophyll content (μg L^−1^), and turbidity (NTU) among the survey stations (St.1-St.12, St.A1-St.A2) in 9:00–12:00, May 30 and August 11, 2017. In addition, we used an acoustic doppler velocimeter (Linquest, USA) to measure the velocity in the study area before and after the reef construction. Before the reef construction (July 30–31 and August 6–7 2007), the current in the study area was dominated by tidal current and was the characteristics of reciprocating flow with a weak tidal residual current. The measured maximum velocity of spring tide was 0.86 m s^−1^ with the flow direction of 252°, and that of neap tide was 0.66 m s^−1^ with the flow direction of 64°. After the reef construction (September 2017), the average velocity of the spring tide and neap tide among survey stations in the study area was 0.28–0.37 m s^−1^*.*

### Statistical analysis

The Shannon–Weaver diversity index *H'*^40^, Pielou's evenness index *J*^41^, Margalef richness index *D*^42^, and Pinkas relative importance index *IRI*^43^ were used to analyze the community diversity, and a cluster analysis was performed to analyze the community structure in different seasons. The calculation formulae were as follows:

$$ H^{\prime} = - \mathop \sum \limits_{i = 1}^{s} p_{i} \log_{2} p_{i} $$

$$ J = H^{\prime}/\ln S $$

$$ D = \left( {S {-}1} \right)/\ln N $$

$$ IRI = \, \left( {N_{i} + W_{i} } \right) \times F_{i} \times {1}0^{{4}} $$

where ${p}_{i}$ is the proportion of total samples number (*N*) belonging to *i*th species; *S* is the total number of species; *N*_*i*_ is the ratio of the species *s* number to the total sample number; *W*_*i*_ is the ratio of the species *i'*s biomass to total samples biomass; *F*_*i*_ is the frequency of occurrence of species *i* at each station. In the cases of *H'* > *3*, *1* ≤ *H'* ≤ *3,* and *0* < *H'* < *1*, the biological community was in a situation of undisturbed, moderately disturbed, and highly disturbed, respectively^44^. The cases of *IRI* > 1000, 100 ≤ *IRI* < 1000, 10 ≤ *IRI* < 100, and *IRI* < 10 indicated dominant, important, common, and rare species, respectively^45^.

Multivariate analysis was performed through hierarchical cluster analysis, which is used to delineate groups of related objects within nonmetric multidimensional scaling (nMDS) ordination space, and nMDS, which is an ordination procedure based on ranked similarity data. Regarding the cluster analysis, the square root of biomass at each station was calculated to stabilize the impact of dominant species in the system. In calculating the Bray–Curtis similarity coefficient matrix, two-dimensional scaling ranking and hierarchical clustering was obtained through the application of nMDS. The confidence of nMDS calculations was evaluated according to the stress coefficient. Stress coefficients show how well the multivariate pattern is represented within lower dimensional space^46,47^.

Finally, abundance biomass curves (ABCs) were used to understand the biological community disturbance degree according to the relative position of the abundance curve and biomass dominance curve in a same coordinate system. If the biomass curve was above the abundance curve, it indicated an “undisturbed” community; if the position of curves were in contrast to each other, it indicated a “serious interference”; if two curves were generally intersecting each other, it meant a “moderate interference”. The ABCs were drawn based on the proportion of the biomass and abundance of each species in the total biomass and abundance of marine organisms in different seasons. The formula was expressed as follows ^48^:

$$ {\text{W}} = \mathop \sum \limits_{{{\text{i}} = 1}}^{{\text{S}}} \frac{{{\text{B}}_{{\text{i}}} - {\text{A}}_{{\text{i}}} }}{{50\left( {{\text{S}} - 1} \right)}} $$

where *S* is the species number, *B*_*i*_ is the biomass cumulative percentage of the species* I*, *A*_*i*_ is the abundance cumulative percentage of the species* i*. The cases of *W* > *0*, *W* < *0,* and *W ≈ 0*, indicate an undisturbed, disturbed, and moderately disturbed community, respectively. The closer the *W* value is to 1, the richness of each species is closer to the similarity, and vice versa for when the *W* value is closer to − 1.

The formulae of the niche width index^40^ and niche overlap index^49^ were as follows:

$$ {\text{B}}_{{\text{i}}} = - \mathop \sum \limits_{{{\text{j}} = 1}}^{{\text{R}}} \left( {{\text{P}}_{{{\text{ij}}}} {\text{ln P}}_{{{\text{ij}}}} } \right) $$

$$ {\text{Q}}_{{{\text{ik}}}} = \mathop \sum \limits_{{{\text{j}} = 1}}^{{\text{R}}} \left( {{\text{P}}_{{{\text{ij}}}} {\text{P}}_{{{\text{kj}}}} } \right)/\sqrt {\mathop \sum \limits_{{{\text{j}} = 1}}^{{\text{R}}} {\text{P}}_{{{\text{ij}}}}^{2} \mathop \sum \limits_{{{\text{j}} = 1}}^{{\text{R}}} {\text{P}}_{{{\text{kj}}}}^{2} } $$

where P_ij_ is the ratio of the ith species abundance at station j to the total abundance at station j; P_kj_ is the ratio of the kth species abundance at station j to the total abundance at station j; B_i_ is the niche width of species i; and R is the total station number. The larger the value of B_i_, the larger the species niche width. Q_ik_ is the ecological overlap index of the species i and k with the value range of 0 and 1. The larger the Q_ik_, the stronger the similarity in resource utilization in the species i and k. The cases of B_i_ ≥ 2.0, 1.0 ≤ B_i_ < 2.0, and B_i_ < 1.0 indicate a wide, medium, and narrow niche species, respectively. The cases of Q_ik_ > 0.7, 0.4 < Q_ik_ < 0.7, and Q_ik_ < 0.4, within the value range of 0.0 to 1.0, indicate a high, median, and low niche overlap degree, respectively^50^. Ecological niche width refers to the sum of the varieties of resources that can be exploited by the organisms. It is an index of resource diversity exploited by the organism. When a species’ ecological niche width is wider, the specialization of this species is smaller (i.e., the species has a wide ecological niche). A narrow niche width indicates that a species is specialized. Niche overlap means that two or more species with a similar niche inhabit the same space and share or compete for the same resource. These statistical analyses were conducted in the software PRIMER 5.0 (Plymouth Institute of Oceanography, Plymouth, UK) and the DPS platform^51^.

## Results

### Species composition, dominant species, and biological community diversity

The 56 species recorded during the survey year belonged to 50 genera, 45 families, and 19 orders, and Table 1 lists the numbers in these categories in each survey month, including control sampling. Among them, there were 35, 28, 19, 23 species in the spring (14 orders, 30 families, and 33 genera), summer (15 orders, 26 families, and 27 genera), autumn (8 orders, 15 families, and 18 genera), and winter (9 orders, 18 families, and 21 genera), respectively. *Palaemon gravieri*, *C. japonica, S. ommaturus*, *H. otakii*, *Pholis fangi*, *S. schlegelii,* and *O. oratoria* were recorded in all seasons; *R. venosa* was present in spring to autumn; other species such as *Asterias amurensis* and *Octopus variabilis* were present in spring, summer, and winter; and *Chaeturichthys stigmatias* and *Alpheus japonicus* were present in spring, autumn, and winter (Table 2). Compared with the oyster reef area, *Philyra platycheir* and *Johnius belangerii* were found only in the control area, and the abundances of *D. edwardsii* (153 vs. 17) and *C. stigmatias* (190 vs. 27) were greater in the control area than in the oyster reef area (Table 2).

**Table 1:** Total number and number by month of order, family, genus, and species in the oyster reef areas and control area in different seasons.

		Order	Family	Genus	Species
Spring	March 2017	9	20	23	26
	May 2017	10	20	20	20
Summer	July 2016	12	18	18	19
	August 2017	11	20	20	21
Autumn	September 2016	9	15	18	19
Winter	December 2016	7	11	11	12
	January 2017	7	16	19	20
Control	July 2017	8	23	26	27
Total		19	45	50	56

**Table 2:** Species composition classified by order and family among seasons and the biomass (unit: g) and abundance (unit: ind.) of each species in the artificial oyster reef areas and control area. The symbol ● means present in this month and season. Values in parentheses represent the niche width index.—means no records. *B* and *N* represent the biomass and number.

Latin name	Common name	Spring			Summer			Autumn		Winter		Control		Oyster reef		Control area	
		March	May		July	August		September		December	January		July	*B*	*N*	*B*	*N*
Aciculata																	
Aphroditidae																	
*Aphrodita talpa*	–	●(0.38)		●						●(0.09)	●(0.33)	●		211.16	11		
Acropomatiformes																	
Lateolabracidae																	
*Lateolabrax japonicus*	Japanese seabass					●(0.03)	●							46.85	1		
Amphilepidida																	
Amphiuridae																	
*Amphiura vadicola*	–	●(0.06)		●										0.50	1		
Clupeiformes																	
Clupeidae																	
*Konosirus punctatus*	Dotted gizzard shad					●(0.03)	●							49.33	1		
Engraulidae																	
*Thryssa kammalensis*	Thryssa kammalensis				●(0.25)	●(0.04)	●							28.48	24		
Decapoda																	
Alpheidae																	
*Alpheus distinguendus*	Forceps snapping shrimp							●(0.57)	●		●(0.06)	●	●(0.12)	12.37	8	11.15	4
*Alpheus japonicus*	Japanese snapping shrimp	●(0.24)	●(0.07)	●				●(0.11)	●	●(0.08)	●(0.14)	●	●(0.63)	14.42	8	77.53	28
Crangonidae																	
*Crangon affinis*	Hakodate sand shrimp	●(0.44)	●(0.11)	●							●(1.96)	●	●(0.02)	98.88	105	0.80	1
Diogenidae																	
*Diogenes edwardsii*	Edward’s hermit crab		●(0.37)	●		●(0.03)	●						●(1.89)	49.21	17	777.31	153
Dorippidae																	
*Dorippe japonica*	–					●(0.09)	●				●(0.24)	●	●(0.60)	25.77	5	201.24	22
Goneplacidae																	
*Eucrate alcocki*	–	●(0.07)		●										8.10	1		
Leucosiidae																	
*Philyra platycheir*	–												●(0.47)			26.30	20
Lysmatidae																	
*Lysmata vittata*	Indian lined shrimp		●(0.96)	●				●(0.12)	●	●(0.11)			●(0.03)	99.07	156	0.34	1
Majidae																	
*Hyastenus diacanthus*	Horn decorator crab										●(0.30)	●		7.64	4		
*Pugettia nipponensis*	–	●(0.34)	●(0.05)	●							●(0.37)	●		91.05	27		
Palaemonidae																	
*Palaemon gravieri*	Chinese ditch prawn	●(1.25)	●(0.45)	●	●(0.21)	●(0.10)	●	●(0.40)	●	●(0.84)	●(2.49)	●	●(2.55)	131.01	153	363.30	353
Pasiphaeidae																	
*Leptochela gracilis*	Lesser glass shrimp	●(0.07)	●(0.07)	●		●(0.43)	●						●(0.40)	36.16	39	8.42	30
Pinnotheridae																	
*Xenophthalmus pinnotheroides*	Blind pea crab										●(0.39)	●		2.55	6		
Portunidae																	
*Charybdis japonica*	Japanese swimming crab	●(0.18)	●(1.57)	●	●(1.70)	●(1.34)	●	●(2.79)	●	●(1.08)	●(0.14)	●	●(2.46)	36,351.92	1180	7229.26	210
*Portunus trituberculatus*	Gazami crab							●(0.11)	●				●(0.28)	73.26	1	734.30	5
Sergestidae																	
*Acetes chinensis*	Northern mauxia shrimp		●(0.11)	●									●(0.07)	0.99	3	0.91	2
Forcipulatida																	
Asteriidae																	
*Asterias amurensis*	North Pacific seastar	●(0.40)	●(1.09)	●	●(0.08)		●			●(0.22)	●(1.44)	●	●(0.08)	11,413.98	246	119.77	3
Gastropoda																	
Naticidae																	
*Neverita didyma*	Bladder moon snail				●(0.03)	●(0.03)	●	●(0.15)	●				●(0.06)	177.39	5	61.90	2
Gobiiformes																	
Gobiidae																	
*Chaeturichthys stigmatias*	Branded goby	●(0.13)		●				●(0.80)	●		●(0.11)	●	●(2.35)	234.12	27	1104.34	190
*Synechogobius ommaturus*	Asian freshwater goby	●(1.45)		●	●(1.48)	●(1.59)	●	●(2.68)	●	●(1.24)	●(3.48)	●	●(0.66)	24,712.41	773	475.19	46
*Tridentiger barbatus*	Shokihaze goby	●(0.52)	●(0.13)	●							●(1.27)	●	●(0.19)	604.68	31	49.16	8
*Tridentiger bifasciatus*	Shimofuri goby	●(0.15)		●										25.30	3		
*Tridentiger trigonocephalus*	Chameleon goby							●(0.05)	●					1.98	1		
Haplosclerida																	
Chalinidae																	
*Haliclona similis*	–		●(0.12)	●										5.73	6		
Myopsida																	
Loliginidae																	
*Loliolus japonica*	Japanese squid					●(0.08)	●							32.20	4		
Nuculida																	
Nuculidae																	
*Ennucula tenuis*	Smooth nutclam		●(0.07)	●										1.11	2		
Neogastropoda																	
Muricidae																	
*Rapana venosa*	Purple whelk	●(0.06)	●(0.87)	●	●(0.46)	●(0.32)	●	●(0.43)	●				●(0.30)	11,094.97	99	870.59	10
Nassariidae																	
*Nassarius siquijorensis*	–	●(0.08)		●							●(0.54)	●		14.11	14		
*Nassarius variciferus*	–	●(0.10)		●									●(0.02)	0.60	2	1.30	1
Octopoda																	
Octopodidae																	
*Octopus variabilis*	Whiparm octopus	●(0.16)	●(0.12)	●	●(0.06)	●(0.08)	●			●(0.13)		●		1935.80	14		
*Amphioctopus fangsiao*	Gold-spot octopus							●(0.46)	●	●(0.15)		●		366.52	14		
Perciformes																	
Cottidae																	
*Trachidermus fasciatus*	Roughskin sculpin										●(0.54)	●	●(0.03)	131.78	6	4.20	1
Hexagrammidae																	
*Hexagrammos agrammus*	Spotty-bellied greenling	●(0.09)		●		●(0.10)	●							116.00	5		
*Hexagrammos otakii*	Fat greenling	●(1.58)	●(0.32)	●	●(0.35)	●(0.03)	●	●(0.37)	●	●(0.74)	●(0.43)	●	●(0.09)	4175.99	126	37.87	3
Pholidae																	
*Pholis fangi*	–	●(0.18)		●	●(0.08)		●	●(1.05)	●		●(0.41)	●	●(0.19)	338.51	47	151.51	7
Platycephalidae																	
*Platycephalus indicus*	Bartail flathead		●(0.04)	●	●(0.08)	●(0.03)	●							318.89	4		
Sciaenidae																	
*Johnius belangerii*	Belanger’s croaker												●(0.76)			601.11	30
Sebastidae																	
*Sebastes schlegelii*	Korean rockfish	●(1.37)	●(1.21)	●	●(1.44)	●(0.50)	●	●(0.75)	●	●(1.40)	●(1.82)	●	●(0.90)	14,528.85	636	51.59	37
Sillaginidae																	
*Sillago sihama*	Silver sillago				●(0.03)		●							9.71	1		
Sparidae																	
*Acanthopagrus schlegelii*	Blackhead seabream					●(0.03)	●	●(0.13)	●					127.81	4		
Stichaeidae																	
*Chirolophis japonicus*	–	●(0.11)		●										116.40	2		
*Ernogrammus hexagrammus*	Six-lined prickleback	●(0.06)		●										27.40	1		
Zoarcidae																	
*Zoarces elongatus*	–	●(0.09)		●										118.60	1		
Pleuronectiformes																	
Cynoglossidae																	
*Cynoglossus joyneri*	Red tonguesole		●(0.05)	●	●(0.10)	●(0.08)	●						●(0.06)	94.97	6	34.20	2
*Cynoglossus robutus*	–				●(0.02)		●							16.39	1		
Paralichthyidae																	
*Paralichthys olivaceus*	Bastard halibut							●(0.33)	●				●(0.03)	162.54	10	122.00	1
Pleuronectidae																	
*Platichthys bicoloratus*	Stone flounder				●(0.23)	●(0.03)	●				●(0.24)	●		246.74	8		
Semaeostomeae																	
Ulmaridae																	
*Aurelia aurita*	Moon jelly				●(0.09)		●							954.95	8		
Stomatopoda																	
Squillidae																	
*Oratosquilla oratoria*	Japanese squillid mantis shrimp	●(0.17)		●	●(0.25)	●(0.08)	●	●(0.27)	●	●(0.18)		●	●(0.19)	537.76	29	62.80	4
Temnopleuroida																	
Temnopleuridae																	
*Temnopleurus hardwickii*	Hardwick’s sea urchin		●(0.04)	●	●(0.08)		●	●(0.07)	●					61.90	5		
Tetraodontiformes																	
Tetraodontidae																	
*Takifugu niphobles*	–		●(0.09)	●	●(0.03)		●							198.07	7		

The seasonally dominant and important species in the artificial oyster reefs were *S. schlegelii* (IRI = 6427.90 and 2189.12), *S. ommaturus* (IRI = 3694.14), *C. japonica* (IRI = 4631.09), *Lysmata vittata* (IRI = 1654.77), *P. gravieri* (IRI = 641.51 and 1789.74), *H. otakii* (IRI = 2995.01 and 272.36), *R. venosa* (IRI = 2044.77), and *Asterias amurensis* (IRI = 2155.25 and 2759.88) in spring; *A. ommaturus* (IRI = 2851.52 and 5156.94), *S. schlegelii* (IRI = 2857.16 and 1553.14), *C. japonica* (IRI = 9377.69 and 7737.86), and *R. venosa* (IRI = 1666.18 and 484.84) in summer; *S. ommaturus* (IRI = 7066.27) and *C. japonica* (IRI = 8148.62) in autumn; and *C. japonica* (IRI = 2691.23), *H. otakii* (IRI = 1040.75), *S. schlegelii* (IRI = 8328.23 and 438.96), *S. ommaturus* (IRI = 5412.43 and 10,117.28), *Crangon affinis* (IRI = 1125.42), *P. gravieri* (IRI = 956.96 and 1241.30), and *A. amurensis* (IRI = 121.34 and 1161.64) in winter. *C. stigmatias* (IRI = 2437.32), *C. japonica* (IRI = 7238.73), *D. edwardsii* (IRI = 1877.99), and *P. gravieri* (IRI = 3251.18) were dominant species in the control area (Table 3). In addition, the IRI of A. amurensis in May (spring) was twice that in January (winter) (2155.25 vs. 1161.64). The IRI of *C. japonica* was similar between summer (9377.69 and 7737.86 for July and August) and autumn (8148.62), and these values were double that in May (4631.09) (Table 3).

**Table 3:** Pinkas relative importance index *IRI* value of different species varying with months of the seasons in the artificial oyster reef areas and control area.

Species	Spring		Summer		Autumn	Winter		Control group
	March	May	July	August	September	December	January	July
*Alpheus japonicus*								221.05
*Aphrodita talpa*	163.53							
*Asterias amurensis*	2759.88	2155.25				121.34	1161.64	
*Aurelia aurita*			158.69					
*Chaeturichthys stigmatias*					292.03			2437.32
*Charybdis japonica*		4631.09	9377.69	7737.86	8148.62	2691.23		7238.73
*Crangon affinis*	102.75						1125.42	
*Diogenes edwardsii*		108.27						1877.99
*Dorippe japonica*								337.71
*Hexagrammos otakii*	2995.01	272.36	279.08		218.93	1040.75		
*Johnius belangerii*								707.62
*Leptochela gracilis*				376.73				
*Lysmata vittata*		1654.77						
*Octopus ocellatus*					161.25			
*Octopus variabilis*	191.55					150.11		
*Oratosquilla oratoria*			148.9					
*Palaemon gravieri*	641.51	1789.74				956.96	1241.3	3251.18
*Paralichthys olivaceus*					128.51			
*Philyra platycheir*								164.96
*Pholis fangi*					543.08			
*Portunus trituberculatus*								224.09
*Pugettia nipponensis*	233.1							
*Rapana venosa*		2044.77	1666.18	485.84	507.16			464.26
*Sebastes schlegelii*	6427.9	2189.12	2857.16	1553.14		8328.23	438.96	350.99
*Synechogobius ommaturus*	3694.14		2851.52	5156.94	7066.27	5412.43	10,117.28	560.46
*Thryssa kammalensis*			182.11					
*Tridentiger barbatus*	222.46						417.87	

Regarding community diversity, the mean H' values were 1.73 ± 0.30 in March and 1.58 ± 0.31 in May, with the highest value (2.16) recorded at St.12 in May and the lowest (1.18) at St.7 in March. The mean *H'* values in summer were 1.41 ± 0.22 in July and 0.99 ± 0.27 in August, with the highest (1.75) at St.4 in July and the lowest (0.65) at St.5 in August. The mean *H'* value in autumn was 1.47 ± 0.27, with the highest (2.06) at St.2 and the lowest (1.18) at St.7. The mean *H'* values in winter were 0.84 ± 0.20 in December and 1.41 ± 0.34 in January, with the highest (1.92) at St.7 in January and the lowest (0.62) at St.10 in December. The mean *H'* value in the control area was 1.97 ± 0.12 in July, with the highest (2.12) at St.A1 and the lowest (1.80) at St.B2 (Table 2). The ranges of *H'* values among stations were 1.18–2.16, 0.65–1.75, 1.18–2.06, and 0.62–1.92 in spring, summer, autumn, and winter, respectively.

For *J*, the mean values in spring were 0.74 ± 0.12 in March and 0.70 ± 0.07 in May, with the highest (0.86) at St.11 and the lowest (0.51) at St.7 in March. The mean values in summer were 0.63 ± 0.06 in July and 0.48 ± 0.06 in August, with the highest (0.70) at St.4 in July and the lowest (0.39) at St.12 in August. The mean value in autumn was 0.71 ± 0.11, with the highest (0.89) at St.3 and the lowest (0.57) at St.7 in September. The mean values in winter were 0.44 ± 0.10 in December and 0.77 ± 0.10 in January, with the highest (0.90) at St.3 in January and the lowest (0.30) at St.10 in December. The mean value in the control area was 0.70 ± 0.03, with the highest (0.76) at St.A3 and the lowest (0.66) at St.B3.

Regarding *D*, the mean values in spring were 2.61 ± 0.64 in March and 1.93 ± 0.63 in May, with the highest (3.49) at St.9 in March and the lowest (1.38) at St.11 in May. The mean values in summer were 1.68 ± 0.26 in July and 1.43 ± 0.49 in August, with the highest (2.17) at St.4 in July and the lowest (0.65) at St.5 in August. The mean value in autumn was 1.74 ± 0.44 in September, with the highest (2.46) at St.2 and the lowest (1.04) at St.3. The mean values in winter were 1.67 ± 0.23 in December and 1.72 ± 0.53 in January, with the highest (2.39) at St.7 and the lowest (0.83) at St.9 in January. The mean value in the control area was 2.86 ± 0.32, with the highest (3.34) at St.A1 and the lowest (2.39) at St.B2 (Table 4).

**Table 4:** Values (mean ± SD and range) of the Shannon–Weaver diversity (*H'*), Pielou’s evenness (*J*), and Margalef richness (*D*) indices among stations (St.1–St.12 and St.A1-St.C3) and seasons (Spring-winter).

	*Stations*		Stations in the oyster reef area													
			St.1	St.2	St.3	St.4	St.5	St.6	St.7	St.8	St.9	St.10	St.11	St.12	The range	Mean ± std
Spring	March 2017	*H'*	–	–	–	–	–	2.01	1.18	1.70	2.09	1.77	1.98	–	1.18–2.09	1.73 ± 0.30
		*J*	–	–	–	–	–	0.81	0.51	0.82	0.75	0.65	0.86	–	0.51–0.86	0.74 ± 0.12
		*D*	–	–	–	–	–	2.77	1.70	1.91	3.49	3.18	2.60	–	1.70–3.49	2.61 ± 0.64
	May 2017	*H'*	–	–	–	–	1.25	–	–	1.42	1.50	–	1.58	2.16	1.25–2.16	1.58 ± 0.31
		*J*	–	–	–	–	0.70	–	–	0.64	0.60	–	0.76	0.78	0.60–0.78	0.70 ± 0.07
		*D*	–	–	–	–	1.40	–	–	1.76	1.98	–	1.38	3.11	1.38–3.11	1.93 ± 0.63
Summer	July 2016	*H'*	1.48	–	–	1.75	–	1.19	1.14	1.48	–	–	–	–	1.14–1.75	1.41 ± 0.22
		*J*	0.64	–	–	0.70	–	0.54	0.58	0.67	–	–	–	–	0.54–0.70	0.63 ± 0.06
		*D*	1.57	–	–	2.17	–	1.57	1.39	1.72	–	–	–	–	1.39–2.17	1.68 ± 0.26
	August 2017	*H'*	–	0.95	–	1.46	0.65	1.06	–	–	–	–	–	0.85	0.65–1.46	0.99 ± 0.27
		*J*	–	0.49	–	0.59	0.47	0.48	–	–	–	–	–	0.39	0.39–0.59	0.48 ± 0.06
		*D*	–	1.12	–	2.06	0.65	1.72	–	–	–	–	–	1.58	0.65–2.06	1.43 ± 0.49
Autumn	September 2016	*H'*	1.49	2.06	1.23	1.66	1.27	1.44	1.18	–	1.41	–	–	–	1.18–2.06	1.47 ± 0.27
		*J*	0.65	0.86	0.89	0.75	0.58	0.69	0.57	–	0.68	–	–	–	0.57–0.89	0.71 ± 0.11
		*D*	1.95	2.46	1.04	2.29	1.79	1.50	1.42	–	1.50	–	–	–	1.04–2.46	1.74 ± 0.44
Winter	December 2016	*H'*	–	–	–	–	–	–	–	–	0.67	0.62	1.07	1.01	0.62–1.07	0.84 ± 0.20
		*J*	–	–	–	–	–	–	–	–	0.37	0.30	0.55	0.52	0.30–0.55	0.44 ± 0.10
		*D*	–	–	–	–	–	–	–	–	1.46	2.06	1.64	1.54	1.46–2.06	1.67 ± 0.23
	January 2017	*H'*	1.64	0.80	1.88	1.53	1.58	1.43	1.92	1.36	0.93	0.97	1.58	1.28	0.80–1.92	1.41 ± 0.34
		*J*	0.75	0.58	0.90	0.73	0.76	0.74	0.83	0.62	0.85	0.89	0.88	0.66	0.58–0.90	0.77 ± 0.10
		*D*	2.35	1.00	1.72	1.75	2.23	1.67	2.39	1.92	0.83	0.96	2.28	1.54	0.83–2.39	1.72 ± 0.53

			The stations of control area													
			St.A1	St.A2	St.A3	St.B1	St.B2	St.B3	St.C2	St.C3					The range	Mean ± std
Control	July 2017	*H'*	2.12	2.04	1.83	2.05	1.80	1.88	2.09	1.93	–	–	–	–	1.80–2.12	1.97 ± 0.12
		*J*	0.73	0.71	0.76	0.76	0.72	0.66	0.75	0.75	–	–	–	–	0.66–0.76	0.7 ± 0.03
		*D*	3.34	3.30	2.84	2.75	2.39	3.02	2.75	2.50	–	–	–	–	2.39–3.34	2.86 ± 0.32

—means no data and the loss of crab pots in the study.

The analysis of seasonal variations revealed that H' and J were more similar among the months of March, May, July, September, January, and July in the control area (from 1.41 ± 0.22 to 1.97 ± 0.12 and from 0.63 ± 0.06 to 0.77 ± 0.10, respectively) compared with August (0.99 ± 0.27 and 0.48 ± 0.06) and December (0.84 ± 0.20 and 0.44 ± 0.10). The values of D were similar in March and July in the control area (2.61 ± 0.64 vs. 2.86 ± 0.32) (Table 4).

### Analysis of community structure and ABCs

**Figure 2:**
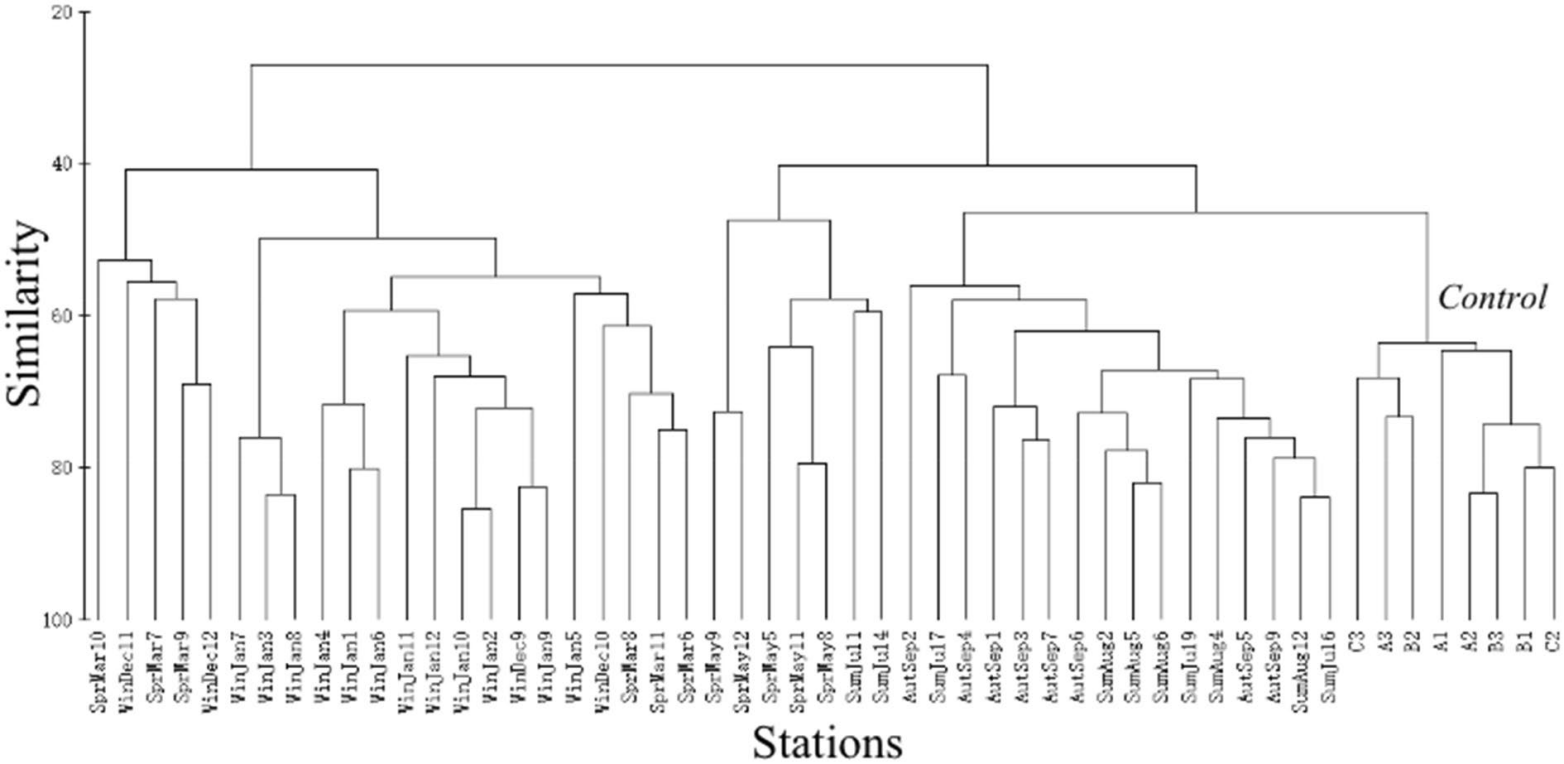
Cluster analysis of the biological communities among sampling stations according to the Bray–Curtis similarity.

**Figure 3:**
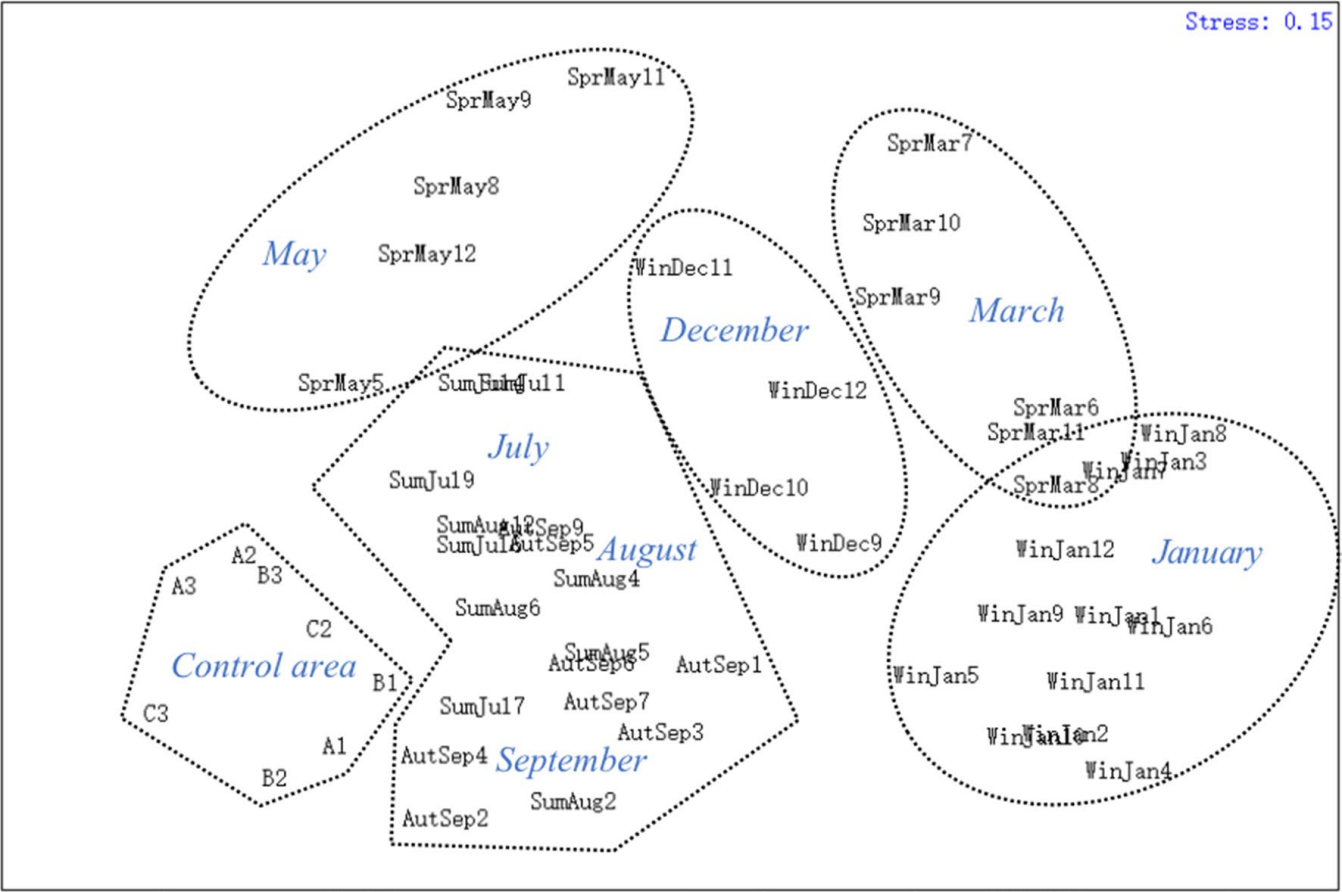
Non-metric multidimensional scaling ordination plot of the biological communities among sampling stations according to the Bray–Curtis similarity. The area is separated into six groups according to months. The stress coefficient value was 0.15 in this study.

**Figure 4:**
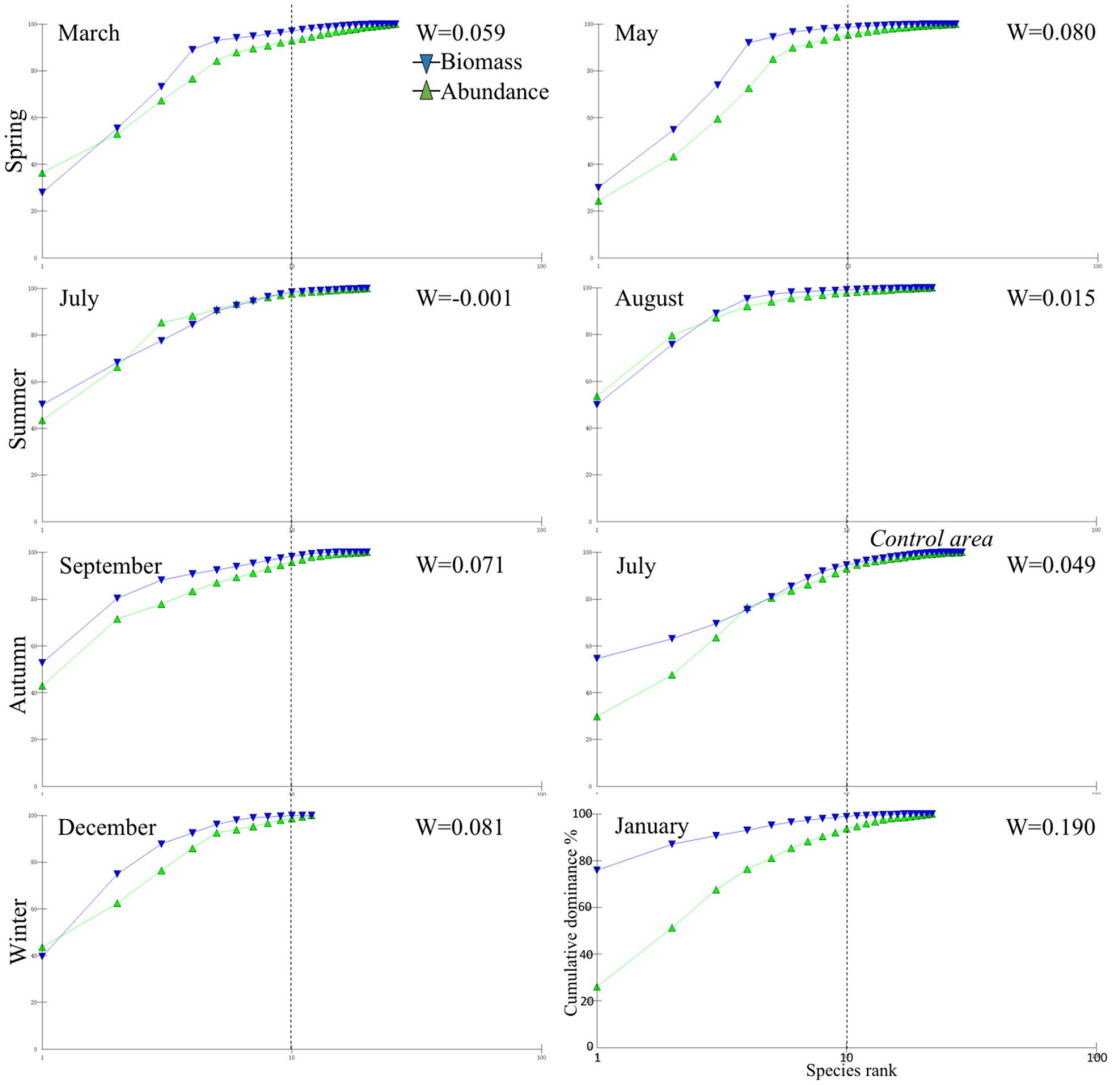
The ABCs of cumulative dominance (unit: %) against species rank varying with months of the seasons. W is the index value of the ABC. The blue inverted triangles denote the biomass curve, and the green triangles denote the abundance curve.

The biological community of the artificial oyster reef area was classified into six groups according to months and seasons: group I (control area), group II (July, August, September), group III (May), group IV (December), group V (March), and group VI (January). These groupings were consistent with the results of nMDS and the clustering analysis (R = 0.824, *P* < 0.01) (Figs. 2, 3). In addition, the stress coefficient of 0.15 shown by the two-dimensional scale sorting of each group indicated a certain explanatory significance for the clustering results of all the stations in different seasons (Figs. 2, 3). The dominance curves of the biomass and abundance all intersected and overlapped in different seasons, indicating that the artificial oyster reef ecosystem experienced “moderate interference” during the survey year. The biomass curves in the reef areas were above the abundance curves in May (spring), September (autumn), and January (winter) as well as in July in the control area, but not in the oyster reef areas in summer, indicating that the oyster reef areas in spring, autumn, and winter experienced low disturbance. In summer, the W value was negative (–0.001 in July) and lower than that in the other three seasons, and the curves intersected in several situations, indicating large variations due to external disturbance (Fig. 4).

### Niche width and niche overlap

The niche width ranges of the marine organisms were 0.06–1.58 in March and 0.02–2.10 in May (spring), 0.03–2.56 in July and 0.02–1.70 in August (summer), 0.05–2.79 in September (autumn), and 0.06–3.48 in January and 0.08–1.40 in December (winter), as well as 0.02–2.55 in July in the control area. The number of species with a wide, medium, and narrow niche width in the oyster reef areas were 0, 5, 21 in March; 1, 4, 22 in May; 0, 3, 17 in July; 1, 2, 20 in August; 2, 1, 16 in September; and 2, 4, 15 in January; 0, 3, 9 in December. They were 3, 1, 28 in July in the control area, (Table 1). The dominant species in each season had a wide niche width, indicating a positive relationship between niche width and *IRI*. The wide niche species were *H. otakii* (1.58) in March and *C. japonica* (1.57) in May; *C. japonica* (1.70) in July and *S. ommaturus* (1.59) in August; *C. japonica* (2.79) and *S. ommaturus* (2.68) in September; *S. ommaturus* (3.48) and *P. gravieri* (2.49) in January; and *P. gravieri* (2.55), *C. japonica* (2.46), and *C. stigmatias* (2.35) in July in the control area (Table 1). The number of species with a wide niche width was greater in autumn and winter than in spring and summer.

Regarding the niche overlap index, 171 pairs were detected. The number of pairs with a high, median, and low niche overlap degree were 35 (20.47%), 42 (24.56%), and 94 (54.97%) in spring, respectively; 44 (25.73%), 33 (19.30%), and 94 (54.97%) in summer; 24 (14.04%), 31 (18.13%), and 116 (67.83%) in autumn; 17 (9.94%), 31 (18.13%), and 123 (71.93%) in winter; and 38 (22.22%), 75 (43.86%), and 58 (22.92%) in the control area. The species pairs with the highest overlap index (1.0) were *Cynoglossus joyneri*-*P. nipponensis* in spring; *T. kammalensis*-*C. robutus*/*Aurelia aurita*, *C. robutus*-*A. aurita*, *O. oratoria*- *T. niphobles*, *O. variabilis*-*T. hardwickii*, *T. niphobles*-*O. variabilis*/*T. hardwickii*, *O. variabilis*-*T. hardwickii*, and *C. joyneri*-*T. hardwickii* in summer; *N. didyma*-*T. hardwickii*/*L. vittata* and *T. hardwickii*-*L. vittata* in autumn; and *Nassarius variciferus*-*Crangon affinis* in the control area. The numbers of pairs with an overlap index of 0.0 were 32 (18.71%), 51 (29.82%), 52 (30.41%), 65 (38.01%), and 26 (15.20%) in spring, summer, autumn, winter, and July in the control area, respectively. The overlap index value of A. ommaturus to other species was highest in summer and winter compared to in spring and autumn. The species *A. ommaturus*, *H. otakii*, and *P. gravieri* occurred at a high frequency and had a higher niche overlap degree compared with other species in winter. The index value of *C. japonica* was the highest, except for in winter, and that of S. schlegelii was the highest in summer compared with the other seasons (Table 5).

**Table 5:** Values of the niche overlap index among different species in the study area from spring to winter (May 2017, July 2016, September 2016, January 2017) and in the control area.

Spring																		
	S1	S2	S3	S4	S5	S6	S7	S8	S9	S10	S11	S12	S13	S14	S15	S16	S17	S18
S2	0.61*																	
S3	0.80**	0.57*																
S4	0.96**	0.64*	0.92**															
S5	0.00	0.12	0.20	0.04														
S6	0.40*	0.69*	0.58*	0.58*	0.00													
S7	0.04	0.51*	0.13	0.18	0.16	0.84**												
S8	0.94**	0.49*	0.90**	0.97**	0.00	0.42*	0.00											
S9	0.03	0.34	0.31	0.11	0.00	0.30	0.00	0.00										
S10	0.01	0.55*	0.07	0.02	0.19	0.03	0.03	0.00	0.12									
S11	0.59*	0.61*	0.72**	0.76**	0.00	0.91**	0.70**	0.67*	0.00	0.00								
S12	0.03	0.34	0.31	0.11	0.00	0.30	0.00	0.00	1.00**	0.12	0.00							
S13	0.06	0.68*	0.17	0.20	0.02	0.87**	0.93**	0.01	0.21	0.28	0.67*	0.21						
S14	0.04	0.75**	0.21	0.19	0.00	0.82**	0.81**	0.00	0.41*	0.45*	0.58*	0.41*	0.96**					
S15	0.02	0.36	0.37	0.11	0.45*	0.26	0.07	0.00	0.89**	0.19	0.00	0.89**	0.20	0.37				
S16	0.59*	0.61*	0.72**	0.76**	0.00	0.91**	0.70**	0.67*	0.00	0.00	1.00**	0.00	0.67*	0.58*	0.00			
S17	0.01	0.60*	0.14	0.05	0.00	0.13	0.00	0.00	0.45*	0.92**	0.00	0.45*	0.32	0.55*	0.40*	0.00		
S18	0.03	0.74**	0.15	0.13	0.25	0.56*	0.65*	0.00	0.12	0.78**	0.44*	0.12	0.80**	0.85**	0.22	0.44*	0.72**	
S19	0.06	0.62*	0.28	0.21	0.00	0.83**	0.74**	0.00	0.66*	0.08	0.53*	0.66*	0.85**	0.88**	0.59*	0.53*	0.29	0.54*

Summer																		
	S1	S2	S3	S20	S4	S21	S5	S22	S23	S6	S24	S7	S8	S25	S9	S26	S10	S11
S2	0.89**																	
S3	0.84**	0.80**																
S20	0.60*	0.82**	0.44*															
S4	0.71**	0.76**	0.63*	0.81**														
S21	0.94**	0.77**	0.74**	0.40*	0.45*													
S5	0.98**	0.79**	0.75**	0.47*	0.63*	0.96**												
S22	0.92**	0.74**	0.74**	0.34	0.40*	1.00**	0.95**											
S23	0.92**	0.74**	0.74**	0.34	0.40*	1.00**	0.95**	1.00**										
S6	0.69*	0.72**	0.52*	0.34	0.28	0.71**	0.67*	0.71**	0.71**									
S24	0.33	0.32	0.16	0.51*	0.82**	0.05	0.32	0.00	0.00	0.00								
S7	0.32	0.29	0.16	0.45*	0.80**	0.05	0.32	0.00	0.00	0.00	1.00**							
S8	0.32	0.29	0.16	0.45*	0.80**	0.05	0.32	0.00	0.00	0.00	1.00**	1.00**						
S25	0.32	0.29	0.16	0.45*	0.80**	0.05	0.32	0.00	0.00	0.00	1.00**	1.00**	1.00**					
S9	0.21	0.53*	0.46*	0.68*	0.45*	0.03	0.00	0.00	0.00	0.00	0.05	0.00	0.00	0.00				
S26	0.13	0.44*	0.00	0.79**	0.32	0.05	0.00	0.00	0.00	0.00	0.07	0.00	0.00	0.00	0.71**			
S10	0.13	0.49*	0.20	0.41*	0.19	0.01	0.00	0.00	0.00	0.64*	0.02	0.00	0.00	0.00	0.43*	0.30		
S11	0.15	0.43*	0.46*	0.22	0.23	0.00	0.00	0.00	0.00	0.50*	0.00	0.00	0.00	0.00	0.50*	0.00	0.85**	
S27	0.15	0.43*	0.46*	0.22	0.23	0.00	0.00	0.00	0.00	0.50*	0.00	0.00	0.00	0.00	0.50*	0.00	0.85**	1.00**

**Autumn**																		
	**S1**	**S2**	**S3**	**S20**	**S35**	**S4**	**S5**	**S24**	**S28**	**S25**	**S10**	**S29**	**S30**	**S31**	**S32**	**S12**	**S13**	**S33**
S2	0.67*																	
S3	0.21	0.55*																
S20	0.37	0.83**	0.54*															
S35	0.03	0.48*	0.09	0.58*														
S4	0.31	0.51*	0.24	0.55*	0.07													
S5	0.10	0.66*	0.65*	0.86**	0.72**	0.17												
S24	0.23	0.53*	0.05	0.82**	0.74**	0.37	0.70**											
S28	0.00	0.19	0.00	0.10	0.70**	0.00	0.33	0.23										
S25	0.00	0.19	0.00	0.10	0.70**	0.00	0.33	0.23	1.00**									
S10	0.01	0.25	0.00	0.11	0.77**	0.00	0.31	0.22	0.95**	0.95**								
S29	0.65*	0.82**	0.25	0.51*	0.39	0.45*	0.36	0.30	0.49*	0.49*	0.46*							
S30	0.04	0.56*	0.62*	0.87**	0.41*	0.66*	0.77**	0.66*	0.00	0.00	0.00	0.19						
S31	0.37	0.77**	0.84**	0.66*	0.21	0.13	0.72**	0.20	0.00	0.00	0.00	0.53*	0.50*					
S32	0.03	0.33	0.96**	0.40*	0.08	0.09	0.61*	0.00	0.00	0.00	0.00	0.00	0.58*	0.69*				
S12	0.03	0.22	0.00	0.04	0.31	0.00	0.00	0.00	0.00	0.00	0.32	0.00	0.00	0.00	0.00			
S13	0.00	0.19	0.00	0.10	0.70**	0.00	0.33	0.23	1.00**	1.00**	0.95**	0.49*	0.00	0.00	0.00	0.00		
S33	0.01	0.21	0.00	0.09	0.29	0.00	0.12	0.05	0.22	0.22	0.35	0.11	0.00	0.00	0.00	0.44*	0.22	
S34	0.03	0.22	0.00	0.04	0.31	0.00	0.00	0.00	0.00	0.00	0.32	0.00	0.00	0.00	0.00	1.00**	0.00	0.44*

Winter																		
	S1	S2	S3	S20	S35	S5	S6	S10	S27	S12	S14	S36	S37	S15	S38	S16	S39	S40
S2	0.14																	
S3	0.45*	0.89**																
S20	0.34	0.09	0.14															
S35	0.00	0.00	0.00	0.23														
S5	0.33	0.00	0.00	0.17	0.00													
S6	0.07	0.00	0.00	0.36	0.86**	0.40*												
S10	0.23	0.51*	0.45*	0.51*	0.34	0.54*	0.65*											
S27	0.14	0.00	0.00	0.12	0.00	0.00	0.06	0.00										
S12	0.14	1.00**	0.89**	0.09	0.00	0.00	0.00	0.51*	0.00									
S14	0.13	0.00	0.00	0.32	0.87**	0.38	0.98**	0.62*	0.11	0.00								
S36	0.18	0.63*	0.57*	0.64*	0.00	0.18	0.16	0.70**	0.00	0.63*	0.10							
S37	0.14	0.00	0.00	0.50*	0.00	0.00	0.09	0.11	0.00	0.00	0.00	0.30						
S15	0.03	0.02	0.02	0.47*	0.25	0.52*	0.59*	0.56*	0.00	0.02	0.51*	0.29	0.19					
S38	0.10	0.00	0.00	0.77**	0.00	0.00	0.08	0.21	0.00	0.00	0.00	0.67*	0.67*	0.31				
S16	0.14	0.00	0.00	0.21	0.00	0.57*	0.35	0.67*	0.00	0.00	0.32	0.40*	0.00	0.13	0.10			
S39	0.00	0.00	0.00	0.49*	0.86**	0.20	0.90**	0.52*	0.00	0.00	0.86**	0.22	0.05	0.68*	0.24	0.05		
S40	0.00	0.00	0.00	0.23	1.00**	0.00	0.86**	0.34	0.00	0.00	0.87**	0.00	0.00	0.25	0.00	0.00	0.86**	
S41	0.06	0.00	0.00	0.20	0.00	0.71**	0.47*	0.59*	0.00	0.00	0.40*	0.13	0.13	0.85**	0.00	0.40*	0.35	0.00

Control area																		
	S19	S17	S24	S2	S10	S5	S3	S11	S14	S35	S1	S4	S33	S28	S6	S12	S36	S42
S17	0.58*																	
S24	0.58*	0.08																
S2	0.91**	0.53*	0.55*															
S10	0.79**	0.73**	0.37	0.86**														
S5	0.70**	0.23	0.19	0.71**	0.39													
S3	0.48*	0.08	0.73**	0.50*	0.38	0.21												
S11	0.66*	0.07	0.87**	0.50*	0.45*	0.16	0.63*											
S14	0.67*	0.16	0.64*	0.70**	0.46*	0.72**	0.70**	0.55*										
S35	0.90**	0.67*	0.47*	0.89**	0.94**	0.48*	0.40*	0.55*	0.44*									
S1	0.88**	0.50*	0.73**	0.90**	0.73**	0.61*	0.77**	0.63*	0.82**	0.76**								
S4	0.73**	0.87**	0.24	0.67*	0.80**	0.36	0.00	0.28	0.19	0.84**	0.51*							
S33	0.60*	0.66*	0.20	0.62*	0.88**	0.11	0.22	0.35	0.10	0.88**	0.45*	0.78**						
S28	0.62*	0.00	0.29	0.53*	0.39	0.65*	0.00	0.50*	0.42*	0.55*	0.31	0.42*	0.35					
S6	0.31	0.38	0.18	0.45*	0.73**	0.00	0.00	0.32	0.18	0.54*	0.23	0.53*	0.67*	0.32				
S12	0.37	0.44*	0.11	0.56*	0.78**	0.06	0.33	0.14	0.12	0.71**	0.38	0.49*	0.87**	0.14	0.63*			
S36	0.44*	0.00	0.00	0.48*	0.19	0.92**	0.00	0.00	0.59*	0.27	0.32	0.20	0.00	0.71**	0.00	0.00		
S42	0.53*	0.90**	0.00	0.38	0.48*	0.23	0.00	0.00	0.00	0.55*	0.38	0.78**	0.50*	0.00	0.00	0.20	0.00	
S15	0.53*	0.90**	0.00	0.38	0.48*	0.23	0.00	0.00	0.00	0.55*	0.38	0.78**	0.50*	0.00	0.00	0.20	0.00	1.00**

S1. *Sebastes schlegelii*; S2. *Charybdis japonica*; S3. *Hexagrammos otakii*; S4. *Rapana venosa*; S5. *Pholis fangi*; S6. *Asterias amurensis*; S7. *Takifugu niphobles*; S8. *Octopus variabilis*; S9. *Platycephalus indicus*; S10. *Palaemon gravieri*; S11. *Cynoglossus joyneri*; S12. *Alpheus japonicus*; S13. *Lysmata vittata*; S14. *Tridentiger barbatus*; S15. *Crangon affinis*; S16. *Pugettia nipponensis*; S17. *Leptochela gracilis*; S18. *Haliclona similis*; S19. *Diogenes edwardsii*; S20. *Synechogobius ommaturus*; S21. *Thryssa kammalensis*; S22. *Cynoglossus robutus*; S23. *Aurelia aurita*; S24. *Oratosquilla oratoria*; S25. *Temnopleurus hardwickii*; S26. *Sillago sihama*; S27. *Platichthys bicoloratus*; S28. *Neverita didyma*; S29. *Octopus ocellatus*; S30. *Acanthopagrus schlegelii*; S31. *Paralichthys olivaceus*; S32. *Tridentiger trigonocephalus*; S33. *Alpheus distinguendus*; S34. *Portunus trituberculatus*; S35. *Chaeturichthys stigmatias*; S36. *Trachidermus fasciatus*; S38. *Xenophthalmus pinnotheroides*; S39. *Nassarius siquijorensis*; S40. *Acetes chinensis;* S41. *Hyastenus diacanthus*; S42. *Nassarius variciferus.*

The symbol ‘**’ means 0.7–1.0, ‘*’ means 0.4–0.7, the *italics* means the median and high niche overlap degree.

### Analysis of oyster reefs and environmental factors

**Figure 5:**
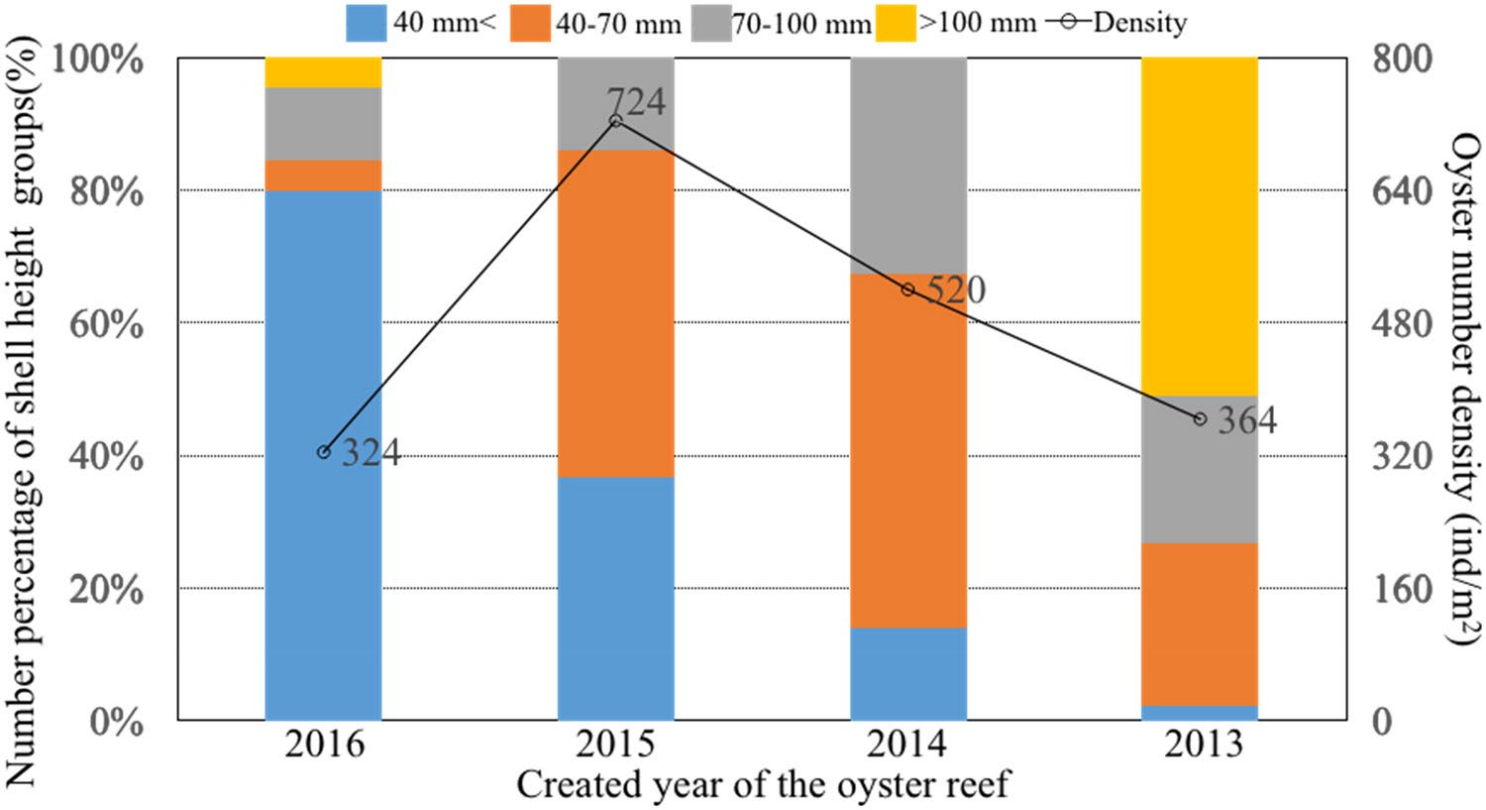
Percentage of oysters collected in July 2016 in shell height groups (%) and oyster density (ind/m^2^) in the reefs created in different years. The left y-axis indicates the height groups < than 40 mm (denoted by blue), 40–70 mm (denoted by reddish brown), 70–100 mm (denoted by gray), and > 100 mm (denoted by yellow). The right y-axis indicates the density variations denoted by hollow circle in the reefs created in 2013, 2014, 2015, and 2016.

In the oyster reefs, 14 species of sessile organisms belonging to 14 genera, 13 families, and 10 orders were found. Among them, 10 and 13 species were found at St.1 (10 genera, 9 families, and 9 orders) and St.2 (13 general, 12 families, and 9 orders), respectively. They had higher biomass and abundance at St.2 compared to St.1 (Table 6). The density of oysters was highest for the oyster reef created in 2015, and then it decreased with increasing reef age (Fig. 5). The percentage of oysters in shell height group < 40 mm decreased with increasing reef age, whereas the percentage of oysters in the > 100 mm group increased with increasing reef age, indicating oyster growth.

**Table 6:** Species composition classified by order and family and the biomass *B* (unit: g) and abundance *N* (unit: ind.) of each species in the oyster reefs in May 2017.

Species		*St.1*		*St.2*	
*Latin name*	*Common name*	*B*	*N*	*B*	*N*
Decapoda					
Lysmatidae					
*Lysmata vittata*	Indian lined shrimp	–	–	1.20	2
Portunidae					
*Charybdis japonica*	Japanese swimming crab	–	–	3.80	4
Xanthidae					
*Actaea savignii*	–	16.00	6	17.60	4
Gobiiformes					
Gobiidae					
*Tridentiger trigonocephalus*	Chameleon goby	4.20	4	8.00	4
Haplosclerida					
Chalinidae					
*Haliclona similis*	–	180.10	46	339.00	66
Mytilida					
Mytilidae					
* Musculus senhousei*	–	1.80	10	0.72	4
*Mytilus edulis*	Blue mussel	13.60	8	13.00	8
Neogastropoda					
Muricidae					
*Rapana venosa*	Purple whelk	2.40	2	33.40	6
Nassariidae					
*Nassarius succinctus*	–	–	–	0.32	4
Ophiurida					
Ophiotrichidae					
* Ophiothrix marenzelleri*	–	5.67	6	–	–
Ostreida					
Ostreidae					
*Crassostrea gigas*	Giant cupped oyster	–	11,933	–	11,173
Pectinida					
Pectinidae					
*Azumapecten farreri*	Farrer’s scallop	51.40	16	113.40	20
Phyllodocida					
Nereididae					
*Nereididae spp.*	–	0.50	2	4.00	12
Sessilia					
Balanidae					
*Balanus spp.*	–	–	–	7.60	4

The value range of water temperature in May and August 2017 were 16.34–18.67 °C and 25.63–27.83 °C respectively in the reef area, and the value difference of mean values among stations in the reef area in May and August was 2 °C. In August, the average value of water temperature in the reef area was 1.07 °C lower than that outside the reef area (26.90 °C against 28.07 °C). In terms of salinity, the average values in May and August were closed (32.52 ppt against 32.25 ppt); the average value in the reef area was a little higher than the outside (32.25 against 31.88 in August). The average value of pH in May and August was similar (8.87 against 8.81), and there was similar in and outside the reef area in August (8.81 against 8.84). The dissolved oxygen value in May was higher than that of August in the reef area (7.91 against 6.03 mg L^−1^); the value of dissolved oxygen outside the reef area was higher than that of reef area in August (6.41 against 6.03 mg L^−1^). The average and range value of total dissolved solids in May was 31.73 and 31.43–31.90 g L^−1^. The average value of chlorophyll in May and August was 6.54 and 5.03 μg L^−1^ respectively with the range of 2.41–9.68 and 3.48–7.38 μg L^−1^; the chlorophyll value outside the reef was higher than that of reef area (5.52 against 5.03) in August. In terms of turbidity, the value in May was far lower than that of August (27.49 against 76.08 NTU), and the value in the reef area was a little lower than outside the reef area (76.08 against 81.26 NTU) (Table 7).

**Table 7:** Month variations in May and August 2017 of the average values of water temperature (unit: °C; *Temp*), salinity (unit: ppt; *Sal*), *pH*, dissolved oxygen (unit: mg L^−1^; *LDO*), total dissolved solids (unit: g L^−1^; *TDS*), chlorophyll (unit: μg L^−1^; *Chl*), and turbidity (unit: NTU; *Turb*) among the survey stations (St.1-St.12, St.A1-St.A2).

May 2017							
*Station*	*Temp*	*Sal*	*pH*	*LDO*	*TDS*	*Chl*	*Turb*
St.1	18.11	32.49	8.83	–	31.70	9.02	63.12
St.2	17.90	32.52	8.89	–	–	8.28	33.04
St.3	17.82	32.53	8.89	–	–	8.39	36.86
St.4	17.99	32.53	8.89	–	31.74	8.58	21.60
St.5	18.64	32.70	8.85	7.89	31.90	9.68	21.19
St.6	18.67	32.69	8.86	7.90	31.90	7.03	17.30
St.7	17.06	32.54	8.89	–	31.73	2.44	12.81
St.8	17.00	32.53	8.86	8.01	31.74	5.45	–
St.9	16.64	32.52	8.88	–	31.74	2.64	34.19
St.11	17.79	32.48	8.85	7.73	31.70	8.00	24.42
St.12	16.34	32.18	8.88	8.03	31.43	2.41	10.40
Average	17.63	32.52	8.87	7.91	31.73	6.54	27.49

August 2017							
*Station*	*Temp*	*Sal*	*pH*	*LDO*	*TDS*	*Chl*	*Turb*
St.1	25.63	32.33	8.79	5.49	–	3.83	–
St.2	27.77	32.32	8.85	6.59	–	6.88	–
St.3	27.78	32.30	8.84	6.62	–	7.21	–
St.4	27.83	32.29	8.84	6.67	–	5.73	84.86
St.5	26.85	32.00	8.81	5.75	–	4.12	–
St.6	26.26	32.34	8.77	5.64	–	4.02	–
St.7	27.45	31.81	8.83	5.90	–	5.38	79.13
St.8	26.83	32.27	8.76	5.96	–	4.81	77.95
St.9	26.47	32.38	8.82	5.87	–	3.48	62.76
St.10	25.98	32.35	8.78	5.62	–	3.58	72.10
St.11	27.50	32.32	8.85	6.48	–	7.38	80.04
St.12	26.44	32.33	8.75	5.73	–	3.95	75.72
Average	26.90	32.25	8.81	6.03		5.03	76.08

*Station*	*Temp*	*Sal*	*pH*	*LDO*	*TDS*	*Chl*	*Turb*
A1	28.11	32.27	8.84	6.60		5.98	81.44
A2	28.03	31.49	8.85	6.22		5.05	81.09
Average	28.07	31.88	8.84	6.41		5.52	81.26

## Discussion

Oysters successfully create vertical complex relief structures that provide microhabitats^52^ for a variety of marine organisms. Fifty-six species were found in the artificial oyster reef areas of this study. Oyster reefs are critical stable habitats for a variety of resident and seasonal resident fishes such as *S. ommaturus*, *C. stigmatias*, *P. fangi*, *H. otakii*, and *S. schlegelii*, economically important crustaceans such as *C. japonica* and *O. oratoria* in different seasons, and molluscs such as *R. venosa* and *O. variabilis*. Similarly, 15 fish, 10 crustacean, and 2 cephalopods species and important resident species including *O. oratoria*, *C. joyneri*, *C. japonica*, *A. hexanema*, *K. punctatus*, and *L. beka* were found in the subtidal wetland with sandy substrate in the Tianjin Dashentang oyster reefs^17^. Quan and Wang (2013) identified significantly greater densities and biomass of living natural intertidal oysters *Crassostrea sikamea* in oyster aquaculture gear than in intertidal oyster reefs in the Xiangshan Bay of Zhejiang Province, China. Greater Pielou evenness and Shannon index values were observed in the intertidal oyster reefs^53^. Yeager et al. (2011) reported that artificial oyster reefs provide a critical refuge for diverse communities of fishes and invertebrates such as blue crabs and striped bass^54^. Gregalis et al. (2009) found higher abundances of small demersal fish and sessile invertebrate species on restored oyster reefs compared with unstructured bottom sediment in coastal Alabama^55^. Ruesink et al.^56^ suggested that the structured habitat constructed by oyster reefs can support a diversity of taxa, including macroalgae, sessile and mobile invertebrate epifauna, infauna, fish, and birds that may be present at decreased numbers or absent in adjacent unvegetated soft-sediment habitats . Dumbauld et al.^57^ found the juvenile Dungeness crab (*Cancer magister*) numbers were enhanced in the oyster shell habitat and thereby compensated for habitat loss caused by dredging.

Additionally, Tolley and Volety (2005) found that crab and fish density, biomass, and diversity were all greater on reefs compared with an unstructured sand bottom at a Florida location^58^. Humphries et al.^59^ reported that the nekton assemblage at oyster reef sites had greater diversity, biomass, and abundance compared with mud bottom sites in Louisiana . It is well-recognized that artificial structures such as artificial oyster reefs can be quite different from those in adjacent rocky areas^60^ and can comprise a diverse assemblage of macroalgae and filter-feeding invertebrates^61^. Powers et al.^62^ verified that the emergent habitat provided by mesh bags led to increasing densities of mobile invertebrates and juvenile fish in the case of on-ground clam culture in the United States compared with the adjacent sand flats and natural seagrass areas . However, in the current study, the total species number and diversity H' of the control area were greater than those of the oyster reef area throughout the year-long study from spring to winter (27 vs. 12–26 and 0.84 ± 0.20 to 1.73 ± 0.30 vs. 1.97 ± 0.12). We speculate that because the control area is very close to the oyster reef area, the boundary between reefs and barren areas can attract more fishes and crustaceans than inside the area. Fish species such as gray snapper and crested goby juveniles feed outside the oyster reef area, exhibiting little diet overlap^54^. The restored oyster reef area might provide a corridor between sheltering and foraging grounds^63^; therefore, the reefs in barren areas have a high heterogeneity, and there are complex interspecies relationships^64^.

The community structure of marine organisms in the artificial oyster reefs in the estuary was more easily influenced by disturbances throughout the year. All of the ABCs intersected and overlapped during the seasons, showing various degrees of disturbances (the shift between wet and dry seasons). In our study area, the natural annual runoff of Luanhe was reduced to zero during the summer (June to August), resulting in salinity changes^65^. In the Loxahatchee Estuary in the USA, the optimal salinity zone for oysters has shifted upstream, resulting in the death and subsequent burial of old oyster reefs^66^. Freshwater releases can flush oyster larvae downstream to locations that have unsuitable substrate and create unfavorable salinity conditions for larval survival in estuarine areas^67^. Oysters in southwest Florida spawn continuously, with peak recruitment occurring from May to November. At that time, large freshwater flows from Lake Okeechobee and conditions during the summer expose oyster larvae and associated organisms to low salinities and flush the larvae downstream to locations with substrates that are not suitable for settlement^67^. Variations in physico-chemical characteristics beneath the oyster habitat could lead to a displacement of large-bodied macrofauna (e.g., large bivalves, heart urchins, brittle stars) and the enhancement of small-bodied disturbance-tolerant opportunistic species (e.g., marine worms and capitellid polychaetes)^61^.

We also detected large seasonal and spatial variations in species composition and community structure. The biological community of the artificial oyster reef areas can be divided according to months. For example, the abundances of transient finfish averaged across all of the reefs over time revealed a strong seasonal pattern, but no obvious interannual pattern related to oyster abundance was reported^68^. Our study area is an important spawning and nursery ground for *S. schlegelii* from spring to summer. They migrate out of the reef areas in autumn when water temperature decreases. This fish’s niche width in summer was lower than that in winter (1.44 vs. 1.82) because it mainly preys on the crustacean species *A. chinensis* in summer^69^. Grabowski reported that for reefs constructed in summer, the development of fish and mobile crustaceans was completed during the next spring–summer season^70^. We also found that the values of *H'*, *J*, and *D* in summer were lower than those in the other three seasons. This result shows that the large biomass and abundance of several dominant species such as *S. ommaturus*, *S. schlegelii*, and *C. japonica* had larger impacts on the community structure in summer.

Artificial oyster reefs provide spatial refuges from predation and can alleviate food limitation^71,72^. For example, the range of niche overlap index values among seasons was 0.0–1.0, showing high heterogeneity among seasons and stations. Crustaceans such as *L. vittata* (117.99 g y^−1^ and 190 ind. y^−1^) in spring and *P. gravieri* (225.57 g y^−1^ and 315 ind. y^−1^) and *C. affinis* (96.77 g y^−1^ and 101 ind. y^−1^) in winter may have a large impact on the community structure due to their high abundances. Oysters can promote pelagic fauna by preventing primary production from entering microbial loops, thus allowing it to pass up the food chain to bottom-feeding crustaceans^11^. The resident crustacean species *C. japonica*, with large biomass and abundance (24,341.89 g y^−1^ and 700 ind. y^−1^), had high niche overlap values with other organisms during the year from spring to autumn. Artificial oyster reefs on mudflats can increase the amount of habitat for these crustaceans, thus further increasing the secondary productivity of the estuary and prey organism foraging^73,74^. There are positive correlations between the diversity and abundance of reef-associated species and oyster shell height and biomass^75^. Reef macroinvertebrate communities respond positively to habitat restoration^76^. The dominance of the lower trophic level consumers might be attributed to the high productivity of the environment^77^. Furthermore, the economically important species *A. fangsiao* (217.42 g y^−1^ and 12 ind. y^−1^), *O. variabilis* (914.00 g y^−1^ and 6 ind. y^−1^), and *R. venosa* (9447.73 g y^−1^ and 85 ind. y^−1^) had large biomass and abundance in this artificial oyster reef, highlighting the value of oyster reef habitats for maintaining high densities of resident species such as mollusks.

Hard substrates on the seabed such as live and dead oysters may provide novel habitats for fouling organisms and associated mobile biota^78^. The aggregation of various fish and crustacean species around the artificial structures, including artificial oyster reefs, is also well-recognized^79,80^. Xu et al.^81^ investigated the community structure of polychaetes in reef and non-reef areas and found that the abundance of carnivore species in the reef area was higher than that in the non-reef area, which might be due to the halo effects of the oyster shell reef. Leguerrier et al. (2004) suggested that oyster aquaculture could increase the food supply to various fishes, which was predicted to be true as the result of increasing meiofaunal production^82^. However, there has been some discussion about the potential for direct negative effects of cultured oysters and mussels on fish population, primarily due to predation on fish eggs and larvae^83^. Dumbauld et al.^25^ did not detect an overall increase in fish richness or abundance adjacent to oysters.

Regarding the development of policies for oyster reef protection, 60–80 fishing boats operate in the open sea of Tianjin Hangu Dashentang each year. Chinese fishermen locally harvested *Volachlamys hirasei* in the 1970s, *Ranapa venosa* in the 1980s, and oyster species such as *Ostrea talienwhanensis*, *Ostrea denselamellosa*, and *C. gigas* in the 1990s–2000s^16^. According to the fishing statistics of Dashentang village, about 100,000 t of living oysters were caught from 1999 to 2006, and 90% of them were *C. gigas* and *O. talienwhanensis*. This fishing behavior led to the rapid decline of the height of living oyster reefs and to the annual increase in the rate of empty shells. This was exacerbated by the sale of attachment substrates required for the survival of oyster larvae at a very low price^16^. Thus, we suggest that it is necessary to set up protection areas for artificial and wild oyster reefs and to implement actions such as quota catch and limits on catch size and period.

## Summary

As an important foraging area and component of the estuarine landscape, artificial oyster reefs created in the barren areas near the Luanhe River Estuary provide important spawning and nursery grounds for a variety of fishes and large mobile crustaceans, and thus have a positive impact on biodiversity in the estuary area. In this study, the dominant species such as resident rocky fishes and large crustaceans appeared to have important impacts on the community structure and diversity of the artificial oyster reefs ecosystem. We found that as oyster reef age increased, the percentage of oysters in the low shell height group (< 40 mm) decreased and that of the high shell height group (> 100 mm) increased. The density of oysters was 324 ind/m^2^ in the oyster reef created in 2016, 724 ind/m^2^ in the oyster reef created in 2015, and 364 ind/m^2^ in the oyster reef created in 2013. Future research should focus on the trade-off between the economic and ecological benefits of artificial oyster reef habitat restoration. A system of ecological assessment indicators for the sustainable management of artificial oyster reefs should be developed. The impact of global climate change with increasing water temperature brings uncertainties regarding the management of oyster reefs, especially in the summer. The species composition and community structure of the artificial oyster reefs within the temperate Luanhe River Estuary should also be compared with those in other temperate and subtropical zones globally.

## Data Availability

The datasets used and/or analysed during the current study available from the corresponding author on reasonable request.

## References

[1] Coen LD, Luckenbach MW (2000). Developing success criteria and goals for evaluating oyster reef restoration: Ecological function or resource exploitation?. Ecol. Eng..

[2] Forrest BM, Keeley NB, Hopkins GA, Webb SC, Clement DM (2009). Bivalve aquaculture in estuaries: Review and synthesis of oyster cultivation effects. Aquaculture.

[3] Peterson CH, Grabowski JH, Powers SP (2003). Estimated enhancement of fish production resulting from restoring oyster reef habitat: Quantitative valuation. Mar. Ecol. Prog..

[4] Zimmerman, R. J., Minello, T. J., Baumer, T. J., & Castiglione, M. C. Oyster reef as habitat for estuarine macrofauna. *NOAA Technical Memorandum*, *NMFS-SEFC-249* 1–16 (1989).

[5] Coen LD, Luckenbach MW, Breitburg DL (1999). The role of oyster reefs as essential fish habitat: A review of current knowledge and some new perspectives. Am. Fish. Soc. Symp..

[6] Bersoza Hernández A, Brumbaugh RD, Frederick P, Grizzle R, Luckenbach MW, Peterson CH, Angelini C (2018). Restoring the eastern oyster: How much progress has been made in 53 years?. Front. Ecol. Environ..

[7] Beck MW, Brumbaugh RD, Airoldi L, Carranza A, Coen LD, Crawford C, Guo X (2011). Oyster reefs at risk and recommendations for conservation, restoration, and management. Bioscience.

[8] Newell RI (1988). Ecological changes in Chesapeake bay: are they the result of overharvesting the American oyster, *Crassostrea virginica*. Underst. Estuary. Adv. Chesapeake Bay Res..

[9] Scarcella G, Grati F, Fabi G (2011). Temporal and spatial variation of the fish assemblage around a gas platform in the northern Adriatic Sea, Italy. Turk. J. Fish. Aquat. Sci..

[10] McAfee D, McLeod IM, Boström-Einarsson L, Gillies CL (2020). The value and opportunity of restoring Australia's lost rock oyster reefs. Restor. Ecol..

[11] Grabowski JH, Brumbaugh RD, Conrad RF, Keeler AG, Opaluch JJ, Peterson CH, Smyth AR (2012). Economic valuation of ecosystem services provided by oyster reefs. Bioscience.

[12] Grabowski JH, Michael FP, Charles HP (2011). Assessing the Long Term Economic Value and Costs of the Crab Hole and Clam Shoal Oyster Reef Sanctuaries in North Carolina.

[13] Adams CM, Mayer LM, Rawson P, Brady DC, Newell C (2019). Detrital protein contributes to oyster nutrition and growth in the Damariscotta estuary, Maine, USA. Aquacult. Environ. Interac..

[14] Ruesink JL, Lenihan HS, Trimble AC, Heiman KW, Micheli F, Byers JE, Kay MC (2005). Introduction of non-native oysters: Ecosystem effects and restoration implications. Annu. Rev. Ecol. Evol. Syst..

[15] Grabowski JH, Peterson CH (2007). Restoring oyster reefs to recover ecosystem services. Ecosyst. Eng. Plants Protists.

[16] Fang EJ, Li WW, Yu J (2007). Sustainable use of live oyster reef in Bohai gulf. Modern Fish. Inf..

[17] Yu QY, Zhang XL, Han XX (2014). Elementary study on the fishery resources conservation and the ecological restoration of the live oyster reef in Dashentang, Tianjin. Mar. Econ..

[18] Rick TC, Reeder-Myers LA, Hofman CA, Breitburg D, Lockwood R, Henkes G, Hines AH (2016). Millennial-scale sustainability of the Chesapeake Bay Native American oyster fishery. PNAS.

[19] McLeod IM, Ermgassen Z, Philine SE, Gillies CL, Hancock B, Humphries A, Wolanski E, Day JW, Elliott M, Ramachandran R (2019). Can bivalve habitat restoration improve degraded estuaries?. Coasts and Estuaries: The Future.

[20] Fitzsimons JA, Branigan S, Gillies CL, Brumbaugh RD, Cheng J, DeAngelis BM, Zu Ermgassen PS (2020). Restoring shellfish reefs: Global guidelines for practitioners and scientists. Conserv. Sci. Pract..

[21] Grabowski JH, Hughes AR, Kimbro DL, Dolan MA (2005). How habitat setting influences restored oyster reef communities. Ecology.

[22] FAO. State of world aquaculture 2006. FAO Fisheries Technical Paper 500. Food and Agriculture Organization of the United Nations, Rome (2006).

[23] FAO. FAO Yearbooks of Fishery Statistics Summary tables. Table B1 World Aquaculture Production by Species Groups (2006).ftp://ftp.fao.org/fifi/STAT/summary/b-1.pdf.

[24] FAO. FAO Yearbooks of Fishery Statistics Summary tables. Table A6 World Aquaculture Production of Fish, Crustaceans, Molluscs, etc., by Principal Species in 2006 (2006). ftp://ftp.fao.org/fifi/STAT/summary/a-6.pdf.

[25] Dumbauld BR, Ruesink JL, Rumrill SS (2009). The ecological role of bivalve shellfish aquaculture in the estuarine environment: a review with application to oyster and clam culture in West Coast (USA) estuaries. Aquaculture.

[26] Costa-Pierce BA, Bridger CJ, Stickney RR, McVey JP (2002). Aquaculture facilities as habitats and ecosystems. Responsible Marine Aquaculture.

[27] Dealteris JT, Kilpatrick BD, Rheault RB (2004). A comparative evaluation of the habitat value of shellfish aquaculture gear, submerged aquatic vegetation and a non-vegetated seabed. J. Shellfish Res..

[28] Murray LG, Newell CR, Seed R (2007). Changes in the biodiversity of mussel assemblages induced by two methods of cultivation. J. Shellfish Res..

[29] Xue C (2016). Extents, type and evolution of Luanhe River fan-delta system, China. Mar. Geol. Quat. Geol..

[30] MacKinnon, J., Verkuil, Y.I., Murray, N. (Eds.) IUCN situation analysis on East and Southeast Asian intertidal habitats, with particular reference to the Yellow Sea (including the Bohai Sea). In Occasional paper of the IUCN species survival commission; IUCN: Gland, Switzerland, **2012**; *47*, pp.1–62.

[31] Xu M, Qi ZL, Liu ZL, Quan WM, Zhao Q, Zhang YL, Liu H, Yang LL (2022). Coastal aquaculture farms for the sea cucumber *Apostichopus japonicus* provide spawning and first-year nursery grounds for wild black rockfish, *Sebastes schlegelii*: A case study from the Luanhe River estuary, Bohai Bay, the Bohai Sea, China. Front. Mar. Sci..

[32] Xu M, Yang XY, Song XJ, Xu KD, Yang LL (2021). Seasonal analysis of artificial oyster reef ecosystems: Implications for sustainable fisheries management. Aquacult. Int..

[33] Shan X, Jin X, Dai F, Chen Y, Yang T, Yao J (2016). Population dynamics of fish species in a marine ecosystem: A case study in the Bohai Sea, China. Mar. Coast. Fish..

[34] Zhang LB, Song XY, Hamel JF, Mercier A, Yang H, Hamel J-F, Mercier A (2015). Aquaculture, stock enhancement, and restocking. Developments in Aquaculture and Fisheries Science.

[35] Fan CF, Pei YD, Wang H, Liu ZG, Wang F, Shang ZW, Che JY, Tian LZ (2007). Correlation of oyster shell form and sediment environment from two buried oyster reefs on the northwest coast of Bohai Bay. Quat. Sci..

[36] Xu M, Qi L, Zhang LB, Zhang T, Yang HS, Zhang YL (2019). Ecosystem attributes of trophic models before and after construction of artificial oyster reefs using Ecopath. Aquacult. Environ. Interac..

[37] Jenkins JA, Bart HL, Bowker JD, Bowser PR, MacMillan JR, Nickum JG (2014). Guidelines for use of fishes in research-revised and expanded. Fisheries.

[38] Torigoe K (1981). Oysters in Japan. J. Sci. Hiroshima Univ...

[39] Wakiya Y (1929). Japanese food oysters. Jpn. J. Zool..

[40] Shannon CE, Weaver W (1948). The Mathematical Theory of Communication.

[41] Pielou C (1969). An Introduction to Mathematical Ecology.

[42] Roughgarden J, May RM, Levin SA (2014). Perspectives in Ecological Theory.

[43] Pinkas L (1971). Food habits of albacore, bluefin tuna and bonito in California waters. Calif. Dept. Fish. Game. Fish. Bull..

[44] Yu S, Wang Y, Han X, Zhang Y, Lai H, Zhang K, Zhang L, Shui B (2021). Changes in community structure and trophic level characteristics of fisheries organisms in Sanmen Bay waters. J. Dalian. Ocean. Univ..

[45] Zhu X, Tang Q (2002). Structuring dominant components within fish community in Bohai Sea system. Studia. Mar. Sinica..

[46] Khalaf MA, Kochzius M (2007). Change in trophic community structure of macrobenthic fauna in subtidal areas of the Yangtze River Estuary in spring. Zool. Res..

[47] Clarke KR, Gorley RN, Somerfield SJ, Warwick RM (2014). Change in Marine Communities: An Approach to Statistical Analysis and Interpretation.

[48] Warwick RM (1986). A new method for detecting pollution effects on marine macrobenthic communities. Mar. Biol..

[49] Hurlbert SH (1978). The measurement of niche overlap and some relatives. Ecology.

[50] Wathne JA, Haug T, Lydersen C (2000). Prey preference and niche overlap of ringed seals *Phoca hispida* and harp seals *P. groenlandica* in the Barents Sea. Mar. Ecol. Prog. Ser..

[51] Tang QY, Zhang CX (2013). Data Processing System (DPS) software with experimental design, statistical analysis and data mining developed for use in entomological research. Insect Sci..

[52] Kuykendall KM, Moreno P, Powell EN, Soniat TM, Colley S, Mann R, Munroe DM (2015). The exposed surface area to volume ratio: Is shell more efficient than limestone in promoting oyster recruitment?. J. Shellfish Res..

[53] Quan WM, Wang YL (2013). Comparisons of benthic macrofauna communities in oyster (*Crassostrea sikamea*) aquaculture gears and adjacent natural oyster reef in Xiangshan Bay of Zhejiang Province, East China. Chin. J. Ecol..

[54] Yeager LA, Layman CA (2011). Energy flow to two abundant consumers in a subtropical oyster reef food web. Aquat. Ecol..

[55] Gregalis KC, Johnson MW, Powers SP (2009). Restored oyster reef location and design affect responses of resident and transient fish, crab, and shellfish species in Mobile Bay, Alabama. Trans. Am. Fish. Soc..

[56] Ruesink JL, Lenihan HS, Trimble AC, Heiman KW, Micheli F, Byers JE, Kay MC (2005). Introduction of non-native oysters: Ecosystem effects and restoration implications. Ann. Rev. Ecol. Evol. Syst..

[57] Dumbauld BR, Visser EP, Armstrong DA, Cole-Warner L, Feldman KL, Kauffman BE (2000). Use of oyster shell to create habitat for juvenile dungeness crab in Washington coastal estuaries: Status and prospects. J. Shellfish Res..

[58] Tolley SG, Volety AK, Savarese M (2005). Influence of salinity on the habitat use of oyster reefs in three southwest Florida estuaries. J. Shellfish Res..

[59] Humphries AT, La Peyre MK, Kimball ME, Rozas LP (2011). Testing the effect of habitat structure and complexity on nekton assemblages using experimental oyster reefs. J. Exp. Mar. Biol. Ecol..

[60] Connell SD (2000). Floating pontoons create novel habitats for subtidal epibiota. J. Exp. Mar. Biol. Ecol..

[61] Hughes DJ, Cook EJ, Sayer MDJ (2005). Biofiltration and biofouling on artificial structures in Europe: The potential for mitigating organic impacts. Oceanogr. Mar. Biol. Annu. Rev..

[62] Powers MJ, Peterson CH, Summerson HC, Powers SP (2007). Macroalgal growth on bivalve aquaculture netting enhances nursery habitat for mobile invertebrates and juvenile fishes. Mar. Ecol. Progress Ser..

[63] Gilby BL, Olds AD, Peterson CH, Connolly RM, Voss CM, Bishop MJ, Schlacher TA (2018). Maximizing the benefits of oyster reef restoration for finfish and their fisheries. Fish. Fish..

[64] Buhl-Mortensen L, Vanreusel A, Gooday AJ, Levin LA, Priede IG, Buhl-Mortensen P, Raes M (2010). Biological structures as a source of habitat heterogeneity and biodiversity on the deep ocean margins. Mar. Ecol..

[65] Li J, Feng P (2007). Runoff variations in the Luanhe River Basin during 1956–2002. J. Geogr. Sci..

[66] Metz JL, Stoner EW, Arrington DA (2015). Comparison of substrates for Eastern oyster (*Crassostrea virginica*) spat settlement in the Loxahatchee River Estuary. Florida. J. Shellfish Res..

[67] Volety AK, Savarese M, Tolley SG, Arnold WS, Sime P, Goodman P, Doering PH (2009). Eastern oysters (*Crassostrea virginica*) as an indicator for restoration of Everglades ecosystems. Ecol. Indic..

[68] Peterson CH (2003). Estimated enhancement of fish production resulting from restoring oyster reef habitat: quantitative valuation. Mar. Ecol. Prog. Ser..

[69] Pan XW, Feng C, Yin ZQ (2020). Analysis of stomach contents of *Sebastes schlegelii* in Tangshan Marine Ranch. Hebei Fish..

[70] Grabowski J. H. (Eds.) The influence of trophic interactions, habitat complexity, and landscape setting on community dynamics and restoration of oyster reefs. PhD thesis, University of North Carolina at Chapel Hill, Chapel Hill, NC; **2002**; pp. 1–155.

[71] Humphries AT, La Peyre MK, Decossas GA (2011). The effect of structural complexity, prey density, and “predator-free space” on prey survivorship at created oyster reef mesocosms. PLoS One.

[72] Buschbaum C, Cornelius A, Goedknegt MA (2016). Deeply hidden inside introduced biogenic structures-Pacific oyster reefs reduce detrimental barnacle overgrowth on native blue mussels. J. Sea. Res..

[73] Blancher EC, Park RA, Clough JS, Milroy SP, Graham WM, Rakocinski CF, Leaf R (2017). Establishing nearshore marine secondary productivity baseline estimates for multiple habitats in coastal Mississippi and Alabama using AQUATOX 3.1 NME for use in the Deepwater Horizon natural resource damage assessment. Ecol. Model..

[74] Grabowski JH, Baillie CJ, Baukus A, Carlyle R, Fodrie FJ, Gittman RK, Sullivan K (2022). Fish and invertebrate use of restored vs. natural oyster reefs in a shallow temperate latitude estuary. Ecosphere.

[75] Luckenbach, M. W., Coen, L. D., Ross Jr, P. G., Stephen, J. A. Oyster reef habitat restoration: Relationships between oyster abundance and community development based on two studies in Virginia and South Carolina. *J. Coastal. Res.*, pp 64–78 (2005).

[76] Searles AR, Gipson EE, Walters LJ, Cook GS (2022). Oyster reef restoration facilitates the recovery of macroinvertebrate abundance, diversity, and composition in estuarine communities. Sci. Rep-UK.

[77] Lassalle G, Lobry J, Le Loc’h F, Bustamante P, Certain G, Delmas D, Niquil N (2011). Lower trophic levels and detrital biomass control the Bay of Biscay continental shelf food web: implications for ecosystem management. Prog. Oceanogr..

[78] Ysebaert T, Hart M, Herman P (2009). Impacts of bottom and suspended cultures of mussels *Mytilus* spp. on the surrounding sedimentary environment and macrobenthic biodiversity. Helgoland Mar. Res..

[79] Relini G, Relini M, Montanari M (2000). An offshore buoy as a small artificial island and a fish-aggregating device (FAD) in the Mediterranean. Hydrobiologia.

[80] Morrisey DJ, Cole RG, Davey NK, Handley SJ, Bradley A, Brown SN, Madarasz AL (2006). Abundance and diversity of fish on mussel farms in New Zealand. Aquaculture.

[81] Xu QZ, Xu Q, Zhang LB, Zhang T, Yang HS (2013). Effect of artificial oyster shell reef on benthic polychaeta community structure in Rongcheng Bay, China. Oceanologia et Limnologia Sinica.

[82] Leguerrier D, Niquil N, Petiau A, Bodoy A (2004). Modeling the impact of oyster culture on a mudflat food web in Marennes-Oléron Bay (France). Mar. Ecol. Progress Ser..

[83] Keeley, N., *et al.* Sustainable Aquaculture in New Zealand: review of the ecological effects of farming shellfish and other non-finfish species. Prepared for the Ministry of Fisheries. *Cawthron Report* 1476. Cawthron Institute, Nelson, New Zealand. 180 pp (2009).

